# Exploring the relationship between HIV Testing frequency and sexual behavior, prevention prophylaxis use among MSM in Southern China

**DOI:** 10.1371/journal.pone.0358578

**Published:** 2026-09-29

**Authors:** Yinxia Liang, Min Wang, Yun Zhang, Jinli Mo, Weiyi Tian, Haimei Huang, Jiawen Zhu, Yangfang Wang, Hongbin Peng, Xia Luo, Ping Cen, Chuanyi Ning

**Affiliations:** 1 The Fourth People's Hospital of Nanning, Guangxi, China; 2 The Second Affiliated Hospital, Guangxi Medical University, Nanning, Guangxi, China; 3 School of Nursing, Guangxi Medical University, Guangxi, China; 4 Key Lab of AIDS Prevention and Treatment, Life Sciences Institute, Guangxi Medical University, Guangxi, China; Centers for Disease Control and Prevention, UNITED STATES OF AMERICA

## Abstract

**Aim:**

This study aimed to explore the association between HIV testing trajectories, sexual behavior characteristics and HIV exposure prevention prophylaxis use, to provide a basis for developing more precise HIV prevention strategies.

**Study design:**

This analysis utilized pseudonymized data from a three-arm, open-label randomized controlled trial (RCT) conducted in Guangxi, China, between 2021 and 2023.

**Methods:**

This prospective cohort study enrolled 327 participants from southern China. Longitudinal data—including HIV testing, sexual risk behaviors, and prophylaxis use (PrEP/PEP)—were collected at baseline and quarterly for one year. Group-based trajectory modeling (GBTM) was used to identify behavioral patterns, and person-years (PY) of follow-up were calculated to estimate incidence densities of PrEP/PEP use and STI occurrence.

**Results:**

A total of 76.45% of men who have sex with men (MSM) received HIV testing less frequently than once every three months, and HIV self-testing (HIVST) was not significantly associated with increased rates of HIV testing (HT). In the HT model, MSM who underwent middle-frequency HT were less likely to report having fewer temporary sexual partners (OR 0.120, 95% CI 0.017–0.85, P = 0.034) and regular sexual partners (OR 0.502, 95% CI 0.267–0.943, P = 0.032). The use of PrEP increased with higher HT frequency, demonstrating a 3.39-fold (95% CI: 1.377–8.843, P = 0.008) increase in the middle frequency group and a 7.335-fold (95% CI: 2.505–21.475, P < 0.001) increase in the high frequency group compared to the low frequency group. PEP use also increased with higher HT frequency, showing a 6.129-fold (95% CI: 1.921–19.553, P = 0.002) increase in the high frequency group compared to the low frequency group.

**Conclusions:**

While frequent HIV testing demonstrates significant associations with increased sexual partner numbers, its role in facilitating consistent use of HIV exposure prevention prophylaxis remains clinically valuable.

## Introduction

HIV prevalence among men who have sex with men (MSM) remains disproportionately high across global populations. The median HIV prevalence among MSM is significantly higher at 7.5%, nearly ten times the global median. Between 2006 and 2022, the share of new HIV diagnoses among MSM in China expanded dramatically from 2.5% to over one-quarter (25.6%) of total cases [1]. Despite ongoing initiatives to manage HIV, factors such as multiple sexual partners, widespread unprotected sex, and recreational substance use have contributed to an increase in new HIV cases among MSM in the country [2]. Consequently, there remains an urgent need to enhance investment in HIV prevention strategies targeting this population.

HIV testing (HT) serves as a fundamental component of prevention strategies by enabling early detection and timely initiation of treatment, thereby reducing transmission risks [3]. Studies demonstrate that regular testing significantly decreases HIV spread within MSM populations [4]. For MSM, a particularly high-risk group, the Centers for Disease Control and Prevention recommends testing at least once a year, with more frequent testing advised for individuals with multiple sexual partners and high levels of sexual activity [5]. However, the popularity and compliance of HT among MSM are not ideal. An Australian study indicated that HIV retest rates among MSM were below 40% after one year and below 20% after six months [6]. Furthermore, a study conducted in Guangzhou, China, revealed that only 44% of MSM were regularly tested for HIV [7]. Despite progress in HT coverage, only 61.2% of Chinese MSM had confirmed their serostatus by 2022, underscoring the need for enhanced testing promotion strategies [8]. HIV self-testing (HIVST) represents an innovative approach to expanding HT coverage [9]. Previous meta-analyses have consistently demonstrated that HIVST doubles the frequency of testing compared to site-based HIV testing (SBHT) [10,11]. The World Health Organization (WHO) recommends HIVST as a strategy to enhance the accessibility of HT for individuals at elevated risk.

Additionally, HT serves as a crucial gateway to additional prevention services, including both pre- and post-exposure prophylaxis (PrEP/PEP) interventions. PrEP and PEP have proven effective in preventing HIV infection in this population and are currently being promoted and implemented in numerous countries in accordance with WHO guidelines [12]. Despite significant efforts to advance HIV prevention services, marginalized groups in China and globally continue to experience inadequate service coverage [13, 14]. Globally, just 50.0% and 58.6% of MSM are informed about and open to using PrEP [14], whereas 51.6% and 6.0% of MSM are familiar with and have taken advantage of PEP[13]. Despite 70% awareness levels documented in a three-year cross-sectional Chinese study, utilization rates for these prevention tools remained below 30% (PrEP) and 25% (PEP) in MSM populations [15]. A meta-analysis from China also pointed out similar results [16]. Therefore, further investigation is warranted to elucidate existing obstacles and enhance service accessibility for PrEP-eligible MSM populations.

This study aimed to evaluate the use of HIV exposure prophylaxis (PrEP/PEP) and, as a secondary objective, the occurrence of STIs among MSM, while analyzing the relationships between these factors and HT frequency trajectories. Additionally, we assessed unprotected sexual intercourse, the number of sexual partners. It is anticipated that this study will provide a scientific basis and practical guidance for optimizing HIV prevention and control strategies, enhancing the compliance and popularity of PEP and PrEP, and informing the development of subsequent research content and methodologies.

## Methods

### Study design and data source

For this research, we compiled pseudonymized data from a three-arm, open-label randomized controlled trial that was conducted in parallel from 2021 to 2023; Baseline recruitment was conducted from September 1, 2021, to February 28, 2022. Participants completed follow-up visits at 3, 6, 9, and 12 months after baseline. The final follow-up was completed by February 28, 2023 (12 months after the last enrolled participant). The maximum follow-up duration was 12 months for all participants [17]. The complete methodology of the study has been detailed in previous publications. In summary, HIV-negative MSM were eligible to participate if they met the following criteria within the six months prior to enrollment: (1) they had engaged in anal intercourse with another male at least once; (2) they received a negative result for syphilis through rapid testing; and (3) they were residents of Guangxi, China, without intentions to leave the area for the subsequent 12 months.

Every participant received HT and was set for follow-up appointments every three months for a maximum duration of one year (until February 2023) to assess medication status in relation to HIV exposure prophylaxis, monitor STI infection status, and evaluate risk behaviors over time. For inclusion in this analysis, participants were required to have completed a behavioral questionnaire during at least one baseline and one follow-up study visit.

### Variables of interest

#### Outcome variable.

The primary outcome measure was the use of HIV exposure prophylaxis, encompassing both PrEP and PEP. Secondary outcomes included the incidence of STIs, the frequency of HT (including both HIVST and SBHT), the number of sexual partners (both regular and casual), and the frequency of unprotected sexual behavior.

#### Exposure variables.

Exposure variables included age, education level, occupation, monthly income, marital status, all of which were collected during the baseline interview. Additionally, data on the use of addictive substances (such as RUSH and Viagra) over the past six months, as well as sexual behavior characteristics—including age at first sexual encounter, number of sexual partners in the past six months, frequency of unprotected sex among MSM during this period, sexual role, positive sexual partners, and incidents of condom rupture—were also recorded.

### Definition of trajectory groups

Longitudinal changes in repeated measures of behaviors related to sexual activity and HIV testing (including HIVST and SBHT) were examined using group-based trajectory modeling (GBTM) [2,18,19]. Details regarding this procedure can be found in the Data Supplement. The quantity of groups (trajectories) and their categorizations for each outcome were extrapolated from prior research concerning MSM sexual behavior and HT behavior, in addition to certain professional standards [18,20]. HIV testing frequency was categorized based on recommendations from multiple international guidelines. The WHO (2019) recommends annual testing for sexually active MSM and 3–6 monthly testing for those at increased risk [21]; the US CDC (2025) recommends annual screening for MSM, with more frequent testing (every 3–6 months) for high-risk individuals [22]; and the IAS-USA (2024) advises 6-monthly HIV testing for PrEP users and 3-monthly testing for frequently exposed individuals [23]. Accordingly, we defined: Low (<1 test/3 months), Medium (1–2 tests/3 months), and High (≥3 tests/3 months).

### Statistical analysis

GBTM employs finite mixture modeling to identify distinct population subgroups characterized by unique trajectories for one or more outcomes of interest [24]. The optimal trajectory group count was determined by simultaneously minimizing Akaike Information Criterion (AIC) and Bayesian Information Criterion (BIC) values while maximizing mean posterior assignment probabilities, the minimum acceptable posterior assignment probability for trajectory group membership was set at ≥0.8 [25–27]. Although our pre-specified minimum group size was 5%, the 4.8% and 4.91% proportions represent slight deviations due to model optimization based on BIC. We conducted sensitivity analyses with alternative model specifications (2-group and 4-group solutions), which confirmed the presence of a distinct high-frequency group (see Supplementary S1 Table). The original 3-group solution was retained because it provided the best fit to the data (lowest BIC) and clinical interpretability. We achieved parsimonious models by omitting polynomial terms (trajectory shapes) that did not reach statistical significance (p < 0.05). STI incidence and PrEP/PEP utilization rates were derived using person-time analysis, expressed as events per 100 person-years (PY) of follow-up. Confidence intervals (CIs) for these rates were calculated using the Poisson distribution, and a Cox regression model was utilized to compare and analyze the occurrence of events.

### Sensitivity analysis

Missing responses resulted in infrequent missing values (<5% for each variable) and were retained unchanged. The trajectory group modeling utilized SAS software version 9.4 (SAS Institute). For the additional analyses and visualizations, R version 4.3.0 was employed alongside the ggplot2 package.

**Ethical approval** for this study (No. 20210173) was granted by the Institutional Review Board at Guangxi Medical University in China. The raw data supporting the findings of this study are not publicly available due to the sensitive nature of the information collected (including sexual behavior and HIV status), which could compromise participant privacy. Data requests may be directed to the Institutional Review Board at Guangxi Medical University (gxmuyfyll@163.com), which will review requests for data access in accordance with the ethical approval granted for this study.

## Results

### Characteristics of study participants

Among the 330 participants enrolled, 327 (99.90%) completed at least one behavioral questionnaire and participated in this analysis (see Table 1). The average age was 23.3 years (standard deviation 11.3), with 181 participants (55.35%) being under 30 years old. Of the participants, 98 (29.97%) were students, and 293 (89.60%) were unmarried. Nearly a quarter of MSM have used addictive drugs in the past 6 months, 9.17% (30/327) of the MSM had ever been infected with STIs. The majority of MSM underwent HT in the past year (85.02%), and 146 MSM utilized HIVST during that period. A total of 256 MSM (78.29%) reported having between 1–5 male sexual partners in the past six months, while 185 (56.41%) MSM engaged in unprotected sexual behavior, and 90 MSM experienced condom breakage during the same period. Furthermore, 43 (13.15%) MSM reported having sexual partners who were HIV-positive, and 90 (27.52%) MSM experienced condom breakage.

**Table 1:** Enrolment characteristics of participants (N = 327).

Variable	Description[*n*(%)]	Variable	Description[*n*(%)]
Age (year)	181 (55.35)	SBHT (past 6 months)	
<30	107 (32.72)	Yes	278 (85.02)
30-40	39 (11.93)	No	49 (14.98)
>40		HIVST (past 6 months)	
Medical insurance		Yes	143 (43.59)
Yes	285 (87.16)	No	185 (56.41)
No	42 (12.84)	STI	
Education		Yes	30 (9.17)
High school or below	73 (22.32)	No	297 (90.83)
College and undergraduate	238 (72.78)	Age of first sexual intercourse (year)	
Graduate students and above	16 (4.90)	<18	74 (22.63)
Employment		18-30	236 (72.17)
Student	98 (29.97)	>30	17 (5.20)
Non-student	229 (70.03)	Number of sexual partners (past 6 months)	
Monthly income (Chinese Yuan)		0	32(9.79)
<1500	59 (18.04)	1-5	256 (78.29)
1500-3000	68 (20.80)	6-30	39 (11.92.)
3001-5000	96 (29.36)	Sex role	
5001-8000	79 (24.16)	Inserter	104 (31.81)
<8001	25 (7.64)	Inserted party	100 (30.58)
Marital status		Inserter/Inserted party	123 (37.61)
Unmarried.	293 (89.61)	Positive sex partner in the past 6 months	
Married.	23 (7.03)	Yes	43 (13.15)
Divorced.	11 (3.36)	No	230 (70.34)
Sexual orientation		Uncertain	54 (16.51)
Gay	221 (67.58)	Condomless anal sex with men (past 6 months)	
Bisexual	84 (25.69)	Yes	182 (55.66)
Uncertain	22 (6.73)	No	145 (44.34)
Biological gender identity		Drug use	
Yes	286 (87.46)	Yes	82 (25.08)
No	41 (12.54)	No	245 (74.92)
Disclosure of sexual orientation			
Yes	82 (25.08)		
No	245 (74.92)		

SBHT: Site-based HIV testing. HIVST: HIV self-testing. STIs: Sexually transmitted infections

### Description of trajectories

We employed a cohort-based trajectory model to analyze HT behaviors, including both HIVST and SBHT, and the number of sexual partners (both casual and fix) and instances of unprotected sexual behavior. Trajectories 1–4 were identified in this analysis (S1 Table). We found three different behavioral trajectories of TH (Fig 1A). Approximately 77.75% of the 327 participants used HT an average of once every three months or less (referred to as “low frequency”). Meanwhile, 17.45% tested an average of twice every three months, with some variation around this average (“middle frequency”), and 4.80% tested more than average of three times every three months (“high frequency”). In terms of SBHT and HIVST, two groups were identified: 82.56% tested less than 0.5 times every three months (low frequency), while 17.44% tested more than once every three months (indicating a gradual increase in frequency). Additionally, 82.46% were tested less than 0.5 times every three months (low frequency), and 17.54% were tested more than twice every three months, showing minimal variation (middle frequency) (Fig 1B and C). In the modeling of sexual partners, three distinct probability trajectories were identified: high (4.91%), multiple sexual partners (16.56%), and low (78.53%) (Fig 2A). As shown in Fig 2(B),As illustrated in Fig 2, a majority of MSM maintained low levels, representing 94.79% of instances with one or no casual sex partners in a three-month period. As shown in Fig 2(D). In the model of unprotected sexual behavior, we found that most MSM (86.12%) engaged in unprotected sexual behavior less than once every three months. A smaller number of MSM (5.99%) exhibited increased unprotected sexual behavior over time, while another small group (7.89%) demonstrated a decrease in unprotected sexual behavior over the same period.

**Fig 1:**
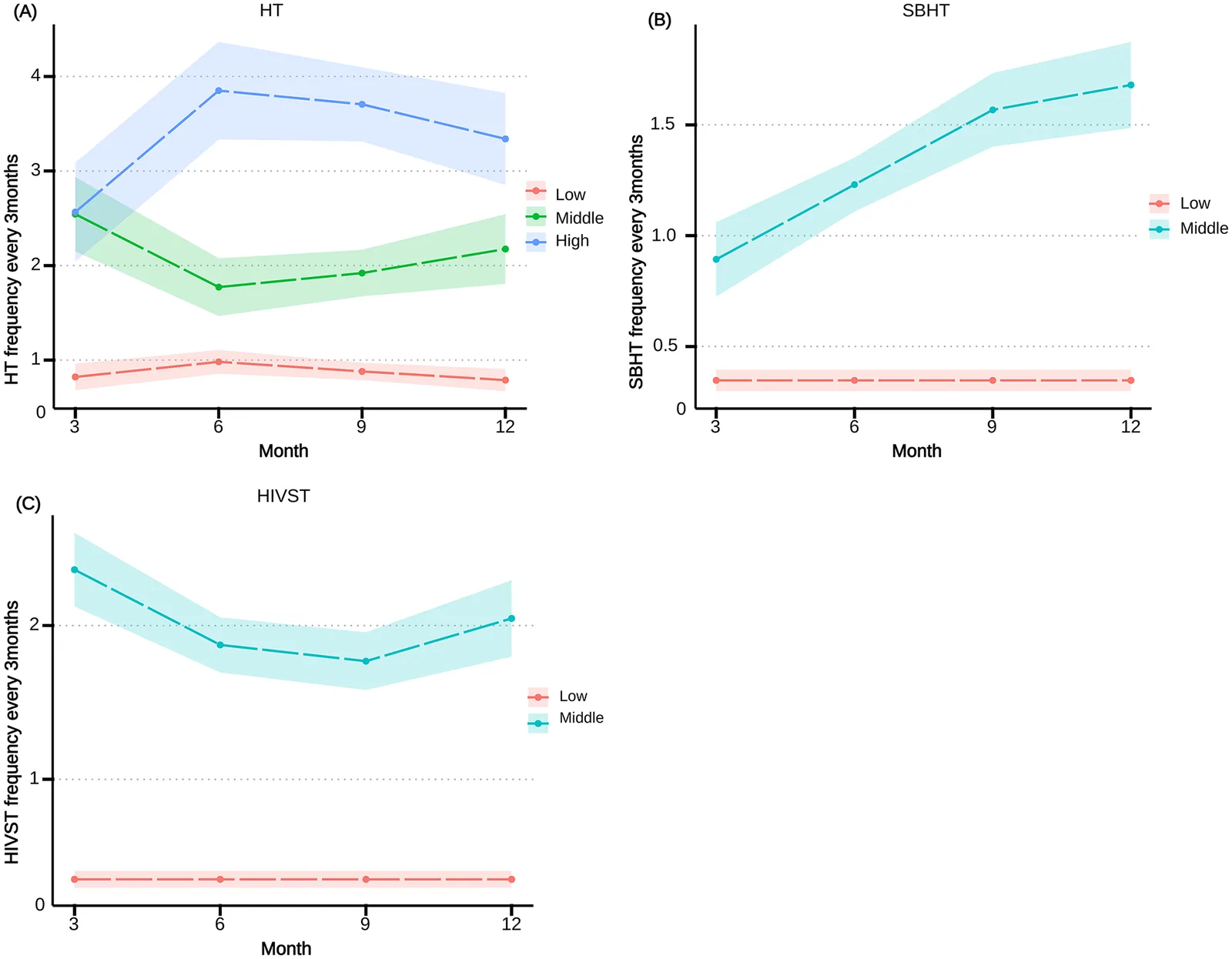
(A) HT: HIV testing. **(B)** SBHT: HIV facility-based testing. **(C)** HIVST: HIV self-testing. Low: < 1 test/3 months. Middle: 1–2 tests/3 months. High: ≥ 3 tests/3 months.

**Fig 2:**
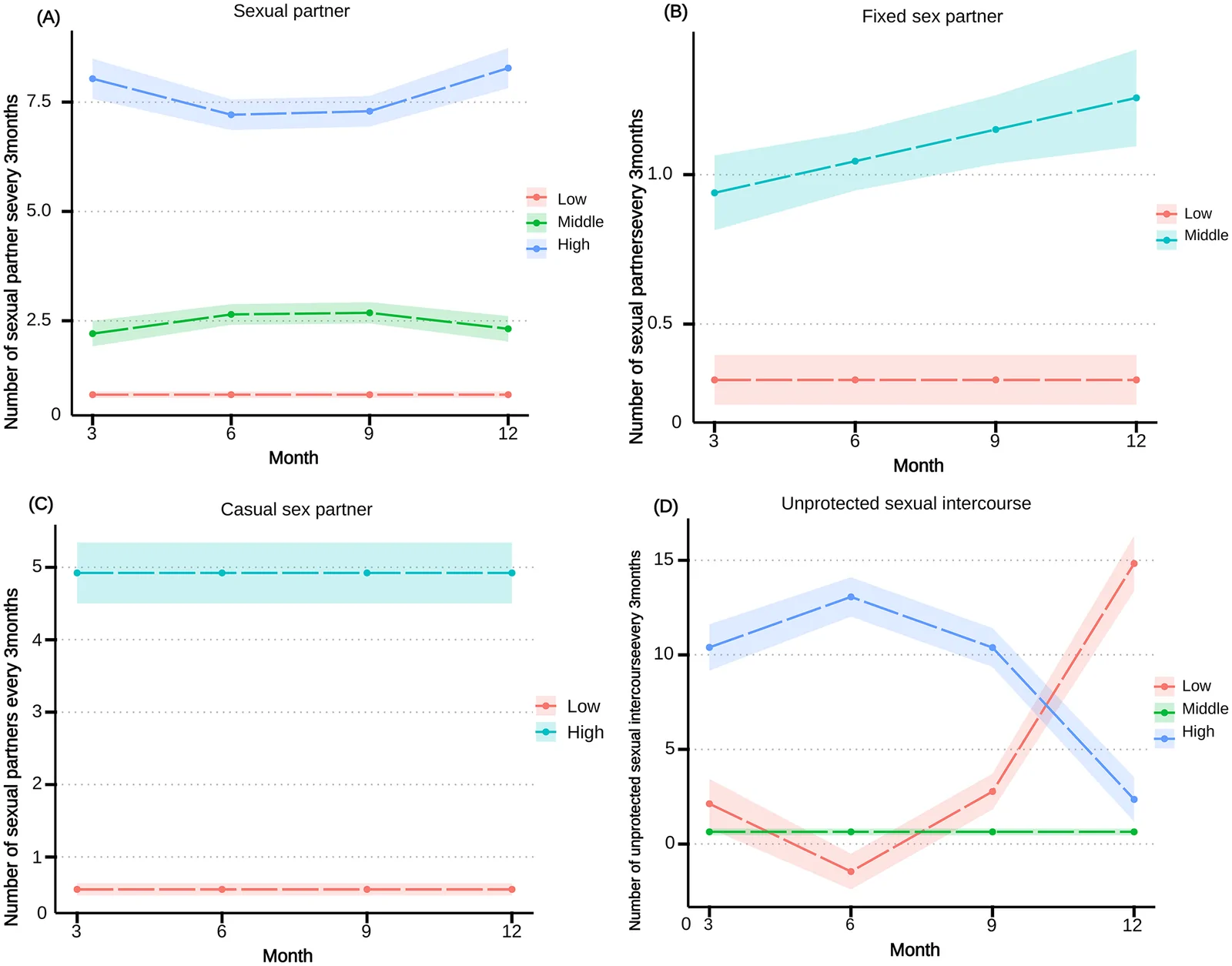
(A) Sexual partner, (B) Fixed sex partner, (C) Casual sex partner: Low: Less than 1 sexual partner every 3 months. Middle: 1-5 sexual partners every 3 months. High: ≥ 6 sexual partners every 3 months. **(D)** Unprotected sexual intercourse: Low: Unprotected sex < 1 times every 3 months. Middle: Unprotected sex 1-5 times every 3 months. High: Unprotected sex ≥ 6 times every 3 months.

### Determinants of HT trajectory membership

In the HT model, participants with “high frequency” HT had sex with men more than 6 times in the past 6 months compared to the other trajectories (p = 0.019). In the SBHT model, Participants with income between “5000 - 8000” RMB were divided into “middle frequency” group (p = 0.022). In the HIVST model, MSM in the middle-frequency group were younger (p = 0.020) and had a higher proportion of students compared to those in the low-frequency group (p = 0.008). Additionally, there was a notable prevalence of substance use (p = 0.007) and unprotected sex (p = 0.038) reported in the last six months. (S2 Table)

### HT and sexual behavior trajectories

In the HT model, MSM who had fewer casual (0.120, 0.017–0.857, P = 0.034) and fix (0.502, 0.267–0.943, P = 0.032) sexual partners were less likely to engage in “moderate frequency” HT compared to “low frequency” trajectories (see Table 2). Similar results were observed in the SBHT model, MSM who had fewer casual (0.105, 0.017–0.670, P = 0.017) sexual partners were less likely to engage in “moderate frequency” (see Table 3)

**Table 2:** Relationship between HIV testing trajectory, number of sexual partners, and unprotected sex trajectory.

Variable	Low HT*	Middle HT*		High HT*		Low SBHT	Middle SBHT^$^		Low HIVST	Middle HIVST^&^	
		aOR (95% CI)	P	OR (95% CI)	P		aOR (95% CI)	P		aOR (95% CI)	P
Trajectory of sexual partners											
Low	1.000	5.887 (0.575 - 60.286)	0.135	0.501 (0.022 - 11.582)	0.666	1.000	1.650 (0.213 - 12.770)	0.632	1.000	11.193 (0.597 - 209.790)	0.106
High		1.000		1.000			1.000			1.000	
Trajectory of fixed sex partners											
Low	1.000	0.120 (0.017 - 0857)	**0.034**	0.267 (0.013 - 5.647)	0.397	1.000	0.105 (0.017 - 0.67)	**0.017**	1.000	0.285 (0.035 - 2.328)	0.206
High		1.000		1.000			1.000			1.000	
Trajectory of casual sex partners											
Low	1.000	0.502 (0.267 - 0.943)	**0.032**	0.485 (0.170 - 1.381)	0.175	1.000	0.680 (0.352 - 1.314)	0.251	1.000	0.680 (0.352 - 1.314)	0.251
High		1.000		1.000			1.000			1.000	
Trajectory of unprotected sexual intercourse											
Low	1.000	0.929 (0.178 - 4.850)	0.930	0.748 (0.055 - 10107)	0.827	1.000	3.306 (0.507 - 21.545)	0.211	1.000	0.521 (0.079 - 3.421)	0.497
Middle		1.068 (0.353 - 3.231)	0.908	0.724 (0.139 - 3.762)	0.700		2.301 (0.500 - 10.580)	0.284		0.952 (0.307 - 2.949)	0.932
High		1.000					1.000			1.000	

*Adjust for frequency of unprotected sex in the past 6 months. $: Adjust for frequency of unprotected sex in the past 6 months. &: Adjustment of occupation, amount of money at your disposal each month, whether you have used drugs in the last 6 months, and have had unprotected sex with men in the last 6 months. Sexual partner: Low: Less than 1 sexual partner every 3 months. Middle: 1–5 sexual partners every 3 months. High: ≥ 6 sexual partners every 3 months. Unprotected sexual intercourse: Low: Unprotected sex < 1 times every 3 months. Middle: Unprotected sex 1–5 times every 3 months. High: Unprotected sex ≥ 6 times every 3 months. HT: HIV testing. SBHT: HIV facility-based testing. HIVST: HIV facility-based testing. Low: HIV testing frequency is less than once every 3 months. Middle: HIV testing frequency 1 times every 3 months. High: HIV testing frequency ≥ 2 times every 3 months.

**Table 3:** STI incidence, PrEP and PEP incidence rates and risk for the assigned sexual and HIV testing trajectory groups.

Probability trajectory	STI incidence rate/100 PY	STI incidence rate ratio	p-Value	PrEP incidence rate/100 PY	PrEP incidence rate ratio	p-Value	PEP incidence rate/100 PY	PEP incidence rate ratio	p-Value
Overall									
(n = 327)	7.65 (5.11, 11.21)			7.03 (4.61, 10.51)			7.65 (5.11, 11.21)		
HIVST									
Low	7.60 (4.76, 11.80)			4.80 (2.69, 8.26)			5.17 (2.96, 8.71)		
Middle	5.85 (1.32, 15.06)	0.668 (0.198, 2.257)	0.516	17.86 (9.34, 30.85)	3.88 (1.70, 8.84)	**0.001**	7.14 (2.31, 18.13)	1.41 (0.46, 4.27)	0.548
SBHT	16.67 (4.41, 42.27)	2.128 (0.630, 7.191)	0.224						
Low				6.57 (4.05, 10.36)			3.28 (1.61, 6.36)		
Middle	7.30 (4.63, 11.22)			9.43 (3.53, 21.42)	1.451 (0.539, 3.908)	0.461	16.98 (8.52, 30.30)	5.370 (2.131, 13.531)	**<0.001**
HT	9.43 (3.53, 21.42)	1.318 (0.495, 3.512)	0.581						
Low				4.00 (2.05, 7.46)			4.00 (2.05, 7.46)		
Middle	6.27 (3.81, 10.04)			13.56 (6.45, 25.53)	3.39 (1.377, 8.843)	**0.008**	6.78 (2.19, 17.27)	1.725 (0.541, 5.500)	0.357
High	14.29 (6.80, 26.78)	2.29 (0.98, 5.31)	**0.053**	27.78 (10.71, 53.59)	7.335 (2.505, 21.475)	**<0.001**	22.22 (73.72, 48.08)	6.129 (1.921, 19.553)	**0.002**

HT: HIV testing. SBHT: HIV facility-based testing. HIVST: HIV facility-based testing. Low: HIV testing frequency is less than once every 3 months. Middle: HIV testing frequency 1 times every 3 months. High: HIV testing frequency ≥ 2 times every 3 months.

### Use of HIV exposure prophylaxis

Overall, among 327 participants, 23 MSM reported using PrEP during follow-up (Table 3). The incidence of PrEP use was 7.03 per 100 PY (95% CI: 4.61, 10.51). In the HT model, PrEP use increased with higher HT frequency, showing a 3.39-fold (95% CI: 1.377–8.843, P = 0.008) increase in the middle frequency group and a 7.335-fold (95% CI: 2.505–21.475, P < 0.001) increase in the high frequency group compared to the low frequency group. In the HIVST model, PrEP use in the middle-frequency HT group was 3.88 times higher than in the low-frequency HIVST group (95% CI: 1.70–8.84, P = 0.004). However, similar results were not observed in the SBHT model. And, among 327 participants, 18 MSM reported using PEP during follow-up (Table 3). The incidence of PEP use was 7.65 per 100 PY (95% CI: 5.11, 11.21). In the HT model, PEP use increased with higher HT frequency, showing a 6.129-fold (95% CI: 1.921–19.553, P = 0.002) increase in the high frequency group compared to the low frequency group. In the SBHT model, PEP use in the middle-frequency group was 5.370 times higher than in the low-frequency SBHT (95% CI: 2.131–13.531, P < 0.001). However, similar results were not observed in the HIVST model.

### Incidence and risk of STI

Overall, 25 cases of STIs were reported among 327 participants (Table 3). The STI incidence was 7.65 per 100 PY (95% CI: 5.11, 11.21). In the HT model, participants in the high-frequency HT group experienced an increased STI incidence of 16.67 (95% CI: 4.41, 42.27). Similar findings were observed in both the SBHT model and the HIVST model. Notably, Cox regression analysis indicated that the incidence of STIs in the middle-frequency HIVST was 2.29 times (95% CI: 0.98, 5.31; P = 0.053) that of the low-frequency HIVST.

## Discussion

This study examines the role of HIVST in expanding HT and investigates the relationships between HT, SBHT, HIVST, and the incidence of unprotected sex, number of sexual partners, and HIV exposure prevention among MSM in a longitudinal cohort in southern China. The results indicate that only a small proportion of MSM engaged in regular HT, defined as testing more than once every three months on average, and the positive impact of HIVST on expanding HT was not observed. Participants in high-frequency HT groups reported a greater number of sexual partners, higher rates of sexually transmitted infections, and increased utilization of HIV exposure prevention measures. These findings highlight the critical need to promote regular HT and enhance high-frequency surveillance among MSM while ensuring timely access to HIV exposure prevention strategies.

To further contextualize these findings, we examined descriptive trends in key outcomes from baseline to 12-month follow-up (Table 3). These data showed sustained HIV testing engagement during follow-up, with 63.30%–68.50% of participants testing at least once per 3 months. However, PrEP and PEP use during follow-up remained low, ranging from 3.98% to 5.50% and 1.83% to 3.06%, respectively. Meanwhile, the proportion of participants reporting unprotected sexual behavior remained relatively stable across follow-up, ranging from 21.71% to 26.60%. Notably, 22 new STI cases were diagnosed during the 12-month follow-up, in addition to 5 participants with STI at baseline. These findings underscore the ongoing STI burden and the need for integrated HIV/STI prevention services, particularly given the sustained level of unprotected sexual behavior observed throughout the follow-up period.

HT is a critical first step in both prevention and treatment efforts. It is essential for determining an individual's HIV status and initiating appropriate treatment [28]. However, in our analysis, 76.45% (250 out of 327) of MSM received HIV test less than once every 3 months. Adherence to regular HIV treatment among MSM may be low [6]. This may be attributed to social prejudice and a lack of awareness about HIV, which can create feelings of shame among many MSM, ultimately leading to resistance to testing and a tendency to avoid it [29–31]. Additionally, insufficient medical resources and limited accessibility to testing services make regular testing inconvenient, reducing the likelihood of participation [32–34]. Technological progress has advanced surveillance methods for MSM, and HIVST has emerged as a crucial complement to traditional HT in this population. In our analysis, only a minority of MSM reported conducting HIVST at least once every three months. Furthermore, HIVST was not significantly linked to increased rates of HT. But compared with SBHT in health care Settings, CDC clinics, and community-based organizations (Cbos), HIVST offers advantages in terms of privacy, confidentiality, and convenience [35], despite the finding in our analysis that over time, Some MSM did have an increase in the frequency of HT, and this increased frequency was associated with an overall rise in HT. But the coverage of SBHT services varies by socioeconomic level. As a complementary approach to SBHT, HIVST can help reduce public health disparities and increase HT coverage among MSM. For instance, digital tools have shown promise in improving HIV testing uptake among MSM. a recent study demonstrated that a chatbot-delivered HIV self-testing service effectively increased testing rates among MSM [36]. Integrating such digital approaches into HIV prevention programs in southern China may complement facility-based testing and help address barriers such as stigma and privacy concerns.

The result indicating that a greater intensity of HT was not linked to alterations in unprotected sexual behavior aligns with earlier observations [37]. However, it was significantly linked to an increase in both casual and regular sexual partners. People who undergo regular HT may show higher awareness of safer sex. For example, studies have found that MSM who test regularly have higher rates of condom use during sex [38,39]. In addition, a high proportion of MSM would choose HT after having experienced multiple sexual partners or unsafe sexual behavior [40]. This finding was consistent across studies examining both HIVST and SBHT. Taken together, these results suggest that the frequency of HT, as well as the choice between self-testing and facility testing, may not significantly influence subsequent sexual behavior. HT practices are influenced by various contexts, including individual, interpersonal, community, and broader social factors. The presence or absence of social support among friends, peers, and the larger communities of gay men plays a significant role in determining men's testing behaviors [41]. The observation that high-frequency HT did not correlate with the incidence of HIV or STIs is significant. Since we did not detect a notable disparity in condom usage between individuals engaging in regular high-frequency HT and those with low-frequency HT, it is not surprising that the study also found no significant difference in HIV or STI incidence between these groups.

Our findings suggest that MSM with high frequency of HT are more likely to use PrEP/PEP compared with MSM with low frequency of HT. Previous studies have indicated that MSM who engage in frequent HT tend to exhibit higher risk awareness [42], possess strong knowledge and acceptance capabilities [43], demonstrate a greater willingness to engage in active prevention, and have improved access to medical resources [44]. Consequently, these individuals are more inclined to accept and utilize PrEP and PEP and maintain consistent usage. After conducting a subgroup analysis, we observed an intriguing phenomenon. MSM who self-tested for HIV with high frequency were more likely to utilize PrEP for prevention compared to those who self-tested infrequently. Similarly, MSM who underwent on-site HT with high frequency exhibited a stronger correlation with the use of PEP than their counterparts who tested on-site less frequently. MSM who frequently self-test for HIV demonstrate a heightened awareness of ongoing risk. Regular self-testing keeps them vigilant regarding the potential for HIV infection. The long-term and continuous use characteristics of PrEP align well with the need for sustained prevention. Additionally, the convenience and privacy associated with self-testing encourage MSM to engage in regular testing and opt for the equally convenient PrEP as a preventive measure [45]. On-site testing typically occurs following high-risk behavior, allowing MSM to receive professional recommendations and guidance. This frequent on-site testing underscores their need for prompt intervention after engaging in high-risk activities. As an emergency preventive measure, PEP effectively addresses this need for immediate prevention. We also observed that although age was correlated with HIVST frequency (younger MSM were more likely to be in the high-frequency self-testing group), we found no significant association between middle-frequency testing trajectories and PEP use. Potential barriers to linking self-testing to PEP for younger MSM include: (1) lack of awareness about PEP availability and access points [46]; (2) perceived low risk despite frequent testing behavior [42]; (3) cost concerns due to the high out-of-pocket expenses for PEP [47]; and (4) fear of disclosure and stigma when seeking PEP at clinics [48]. These barriers highlight the need for integrated interventions that combine HIV self-testing with PEP education and referral services. Future interventions should focus on integrating PEP education into HIV self-testing platforms (e.g., app-based referral systems) and establishing rapid, low-barrier pathways from self-testing to PEP services, particularly for younger MSM who demonstrate high risk awareness through frequent self-testing. HT and HIV exposure prevention are complementary in HIV prevention strategies. Especially the potential of HIVST and HIV pre-exposure prophylaxis among MSM, future research should continue to explore the integration model of the two to improve the effectiveness of HIV prevention and treatment.

### Limitations

This research has multiple limitations. First, the study period (September 2021 to February 2023) overlapped with COVID-19 control measures in China. Although the study sites did not experience strict lockdowns during recruitment, local mobility restrictions and temporary clinic closures may have affected access to HIV testing and PrEP/PEP services for some participants. This could have led to an underestimation of HIV testing frequency and prevention service uptake. Nonetheless, readers should interpret the findings with consideration of this unique pandemic context. Second, participants were solely selected from Guangxi, China, which could result in limited representativeness and generalizability. In particular, our sample overrepresents younger, more educated MSM (mean age 23.3 years; 29.97% students). In comparison, a cohort study in Zhuhai reported that MSM aged 18–24 accounted for 23.18%, with students comprising only 6.62% [49]. A 28-province study (N = 2,618) reported 26.90% aged <25 years and 18.80% students [50], and a Northwest China study reported 16.3% aged 18–25 years with <8.1% students [51]. These comparisons suggest that our sample is significantly younger and more student-dominated than many other Chinese MSM cohorts, limiting generalizability to older MSM and non-student populations. Future studies should include more diverse age and occupational groups to validate our findings.Third, the results interpretation relies on self-reported questionnaires, potentially introducing biases related to reporting and recall. Moreover, the MSM group exhibits a high sensitivity concerning personal privacy and sexual behavior, which might result in hesitance to provide accurate information or engage in certain assessments during the study. Finally, our analysis employed GBTM to identify distinct testing trajectories and Cox regression to examine group differences in outcomes. This approach was chosen to address our primary research question of characterizing heterogeneity in testing patterns. However, we did not apply mixed-effects or GEE models to formally test within-person change over time. While our descriptive comparisons of baseline and follow-up outcomes provide some indication of overall change, formal growth curve models would offer more rigorous estimates of individual-level trajectories. Future studies should consider applying such methods to further characterize within-person changes in HIV testing and prevention behaviors.

## Conclusion

Overall, while a high frequency of HT is associated with having multiple sexual partners and the occurrence of STIs, regular HT can facilitate the use of HIV prevention medications. Notably, PrEP use was more strongly associated with HIV self-testing, while PEP use was more closely linked to facility-based testing. These findings suggest that HIVST may be an effective entry point for PrEP implementation, whereas facility-based testing remains important for PEP access. Differentiated service delivery models should be developed to leverage the unique synergies between each testing modality and its corresponding prevention method.

## Supporting information

S1 Table Model fit assessment indicators for HIV testing and sexual partner/behavior trajectories in MSM. (DOCX)

S2 Table Trajectory group characteristics of HIV testing patterns among MSM. (DOCX)

S3 Table Descriptive changes in HT, HIVST, SBHT, PEP/PEP, unprotected sexual behavior from baseline to 12-month follow-up. (DOCX)
