# Supplementary material for: Exploring the relationship between HIV Testing frequency and sexual behavior, prevention prophylaxis use among MSM in Southern China
Source: PLoS One. 2026 Sep 29;21(9):e0358578. doi: 10.1371/journal.pone.0358578 (PMC13623135; doi:10.1371/journal.pone.0358578)
Supplement: Supplementary file 1 [file pone.0358578.s001.docx]

**sTable 1 Model Fit Assessment Indicators for HIV Testing and Sexual Partner/Behavior Trajectories in MSM**

| **Group** | ***Avepp(%)*** | ***OCC*** | ***P_j_(%)*** | ***π_j_(%)*** | ***BIC^#2^*** | ***△BIC^#2^*** | ***E_k_*** |
| --- | --- | --- | --- | --- | --- | --- | --- |
| **HIV testing** | | | | | | | |
| ***1Group (0 )*** | 100.00 |  | 100.00 | 100.00 | -1890.88 |  | 0.000 |
| ***2Group (0 0 )*** | 95.51-94.60 | 4.0-93.1 | 87.42-12.58 | 84.18-15.82 | -1786.22 | 104.65 | 0.846 |
| ***3Group (2 2 2 )*** | 94.30-83.83-95.15 | 4.7-24.5-389.4 | 80.37-15.34-4.29 | 77.75-17.45-4.80 | -1788.57 | -2.35 | 0.832 |
| ***4Group (0 0 2 2 )*** | 83.94-90.45-94.84-94.60 | 19.5-3.9-597.2-342.7 | 16.56-76.38-2.45-4.60 | 21.11-71.05-2.98-4.86 | -1774.11 | 14.47 | 0.810 |
|  |  |  |  |  |  |  |  |

**HIV self-testing**

| ***1Group (3 )*** | 100.00 |  | 100.00 | 100.00 | -1805.57 |  | 0.000 |
| --- | --- | --- | --- | --- | --- | --- | --- |
| ***2Group (0 2 )*** | 97.58-91.25 | 8.8-47.5 | 82.46-17.54 | 82.00-18.00 | -1656.68 | 148.89 | 0.875 |
| ***3Group (0 1 2 )*** | 96.64-89.77-93.81 | 7.3-50.6-265.6 | 81.54-13.54-4.92 | 79.81-14.79-5.40 | -1617.87 | 38.81 | 0.892 |
| ***4Group (2 2 2 2 )*** | 96.39-88.09-89.91-98.22 | 7.8-48.7-124.2-2063.4 | 79.08-12.62-5.85-2.46 | 77.50-13.20-6.69-2.61 | -1596.83 | 21.04 | 0.904 |
| **Site-based HIV testing** | | | | | | | |
| ***1Group (2 )*** | 100.00 |  | 100.00 | 100.00 | -1448.06 |  | 0.000 |
| ***2Group (0 1 )*** | 96.51-89.28 | 5.8-39.5 | 83.74-16.26 | 82.56-17.44 | -1344.48 | 103.58 | 0.840 |
| ***3Group (0 1 0 )*** | 95.86-85.72-98.92 | 7.5-20.9-4129.8 | 75.46-22.39-2.15 | 75.50-22.34-2.16 | -1308.36 | 36.12 | 0.858 |
| ***4Group (1 0 1 1 )*** | 86.69-96.50-91.80-99.80 | 24.8-9.4-367.0-31421.8 | 21.17-74.54-2.76-1.53 | 20.83-74.67-2.96-1.54 | -1302.63 | 5.73 | 0.894 |
| **Sexual partner** | | | | | | | |
| ***1Group (3 )*** | 100.00 |  | 100.00 | 100.00 | -2472.44 |  | 0.000 |
| ***2Group (0 2 )*** | 99.70-100.00 | 18.1-653512641.7 | 95.09-4.91 | 94.81-5.19 | -1952.64 | 519.80 | 0.985 |
| ***3Group (0 2 2 )*** | 95.44-93.08-99.92 | 6.6-58.1-24436.9 | 78.53-16.56-4.91 | 76.09-18.78-5.12 | -1858.07 | 94.57 | 0.888 |
| ***4Group (0 2 2 2 )*** | 94.79-78.66-82.45-99.96 | 6.2-33.5-40.9-51878.8 | 77.61-8.28-9.20-4.91 | 74.65-9.92-10.31-5.13 | -1863.26 | -5.20 | 0.857 |
| **sTable 1 Model Fit Assessment Indicators for HIV Testing and Sexual Partner/Behavior Trajectories in MSM** | | | | | | | |
| **Fixed sex partner** | | | | | | | |
| **Group** | ***Avepp(%)*** | ***OCC*** | ***P_j_(%)*** | ***π_j_(%)*** | ***BIC^#2^*** | ***△BIC^#2^*** | ***E_k_*** |
| ***1Group (3 )*** | 100.00 |  | 100.00 | 100.00 | -1370.60 |  | 0.000 |
| ***2Group (0 1 )*** | 89.63-81.10 | 4.7-7.9 | 64.72-35.28 | 64.68-35.32 | -1311.12 | 59.48 | 0.582 |
| ***3Group (0 0 2 )*** | 91.35-86.53-99.91 | 7.9-9.0-115940.0 | 56.44-42.64-0.92 | 57.29-41.72-0.99 | -1235.28 | 75.83 | 0.773 |
| ***4Group (0 0 2 0 )*** | 385.72--- | --- | 49.08-8.21-0.99-41.72 | 78.80--- | -1242.37 | -7.09 | 0.650 |
| **Casual sex partner** | | | | | | | |
| ***1Group (0 )*** | 100.00 |  | 100.00 | 100.00 | -2363.53 |  | 0.000 |
| ***2Group (0 0 )*** | 99.59-97.18 | 14.1-596.9 | 94.79-5.21 | 94.55-5.45 | -2186.49 | 177.04 | 0.975 |
| ***3Group (0 0 1 )*** | 90.55-97.78-99.38 | 95.1-5.5-8075.9 | 7.98-90.18-1.84 | 9.16-88.91-1.94 | -2157.50 | 28.99 | 0.933 |
| ***4Group (2 0 2 2 )*** | 90.78-98.34-100.00-96.91 | 90.9-8.1-334632686604-2497.7 | 9.20-88.65-0.92-1.23 | 9.77-88.03-0.96-1.24 | -2005.56 | 151.94 | 0.954 |

**Unprotected sexual intercourse**

| ***1Group (0 )*** | 100.00 |  | 100.00 | 100.00 | -3410.61 |  | 0.000 |
| --- | --- | --- | --- | --- | --- | --- | --- |
| ***2Group (3 3 )*** | 99.23-98.15 | 8.9-770.3 | 94.17-5.83 | 93.56-6.44 | -3238.24 | 172.37 | 0.966 |
| ***3Group (2 0 2 )*** | 93.81-98.62-97.75 | 238.1-11.5-508.0 | 5.83-86.81-7.36 | 5.99-86.12-7.89 | -3104.56 | 133.68 | 0.953 |
| ***4Group (2 0 0 2 )*** | 91.69-97.80-88.53-97.50 | 187.0-8.6-133.0-713.3 | 5.52-84.97-4.60-4.91 | 5.57-83.76-5.48-5.19 | -3100.01 | 4.55 | 0.937 |
| ***Note: Good model fit indicated by (1)Avepp(Average posterior probability)greater than 0.7 for each group,(2)P_j_(Posterior probability of group membership) greater than 5%,(3)Close correspondencePj and πj(Probability of group membership),(4) BIC(Bayesian information criterion) close to 0,(5)The large △BIC(BIC difference between complex and simple models) the more complex models are accepted, (6)OCC(Odds of Correct Classification) greater than 5, (7)E_k_ (Relative entropy) greater than 0.8.*** | | | | | | | |
