# Supplementary material for: Exploring the relationship between HIV Testing frequency and sexual behavior, prevention prophylaxis use among MSM in Southern China
Source: PLoS One. 2026 Sep 29;21(9):e0358578. doi: 10.1371/journal.pone.0358578 (PMC13623135; doi:10.1371/journal.pone.0358578)
Supplement: Supplementary file 2 [file pone.0358578.s002.docx]

| **sTable 2 Trajectory Group Characteristics of HIV Testing Patterns Among MSM** | | | | | | | | | | | | | | |
| --- | --- | --- | --- | --- | --- | --- | --- | --- | --- | --- | --- | --- | --- | --- |
| **Variable** | **Allcount** | **HT** | | |  |  | **SBHT** | |  |  | **HIVST** | |  |  |
|  |  | Low (n=253) | Middle (n=56) | HigH (n=17) | *χ^2^/** | P | Low (n=273) | Middle (n=53) | *χ^2^/** | P | Low (n=271) | Middle (n=56) | *χ^2^/** | P |
| **Age (year)** |  |  |  |  |  |  |  |  |  |  |  |  |  |  |
| <30 | 181 (55.35) | 138(54.55) | 32(57.14) | 11(64.71) | 0.881 | 0.927 | 149(54.58) | 32(60.38) | 0.636 | 0.728 | 141(52.03) | 40(71.43) | 7.801 | **0.020** |
| 30-40 | 107 (32.72) | 84(33.20) | 18(32.14) | 4(23.53) |  |  | 91(33.33) | 15(28.30) |  |  | 97(35.79) | 10(17.86) |  |  |
| >40 | 39 (11.93) | 31(12.25) | 6(10.71) | 2(11.76) |  |  | 33(12.09) | 6(11.32) |  |  | 33(12.18) | 6(10.71) |  |  |
| **Medical insurance** |  |  |  |  |  |  |  |  |  |  |  |  |  |  |
| Yes | 285 (87.16) | 216(85.38) | 52(92.86) | 16(94.12) | 3.070 | 0.215 | 237(86.81) | 47(88.68) | 0.138 | 0.711 | 238(87.82) | 47(83.93) | 0.629 | 0.428 |
| No | 42 (12.84) | 37(14.62) | 4(7.14) | 1(5.88) |  |  | 36(13.19) | 6(11.32) |  |  | 33(12.18) | 9(16.07) |  |  |
| **Education** |  |  |  |  |  |  |  |  |  |  |  |  |  |  |
| High school or below | 73 (22.32) | 56(22.13) | 13(23.21) | 4(23.53) | — | 0.593* | 62(22.71) | 11(20.75) | 0.983 | 0.612 | 64(23.62) | 9(16.07) | 3.478 | 0.176 |
| College and undergraduate | 238 (72.78) | 186(73.52) | 40(71.43) | 11(64.71) |  |  | 199(72.89) | 38(71.70) |  |  | 196(72.32) | 42(75.00) |  |  |
| Graduate students/above | 16 (4.89) | 11(4.35) | 3(5.36) | 2(11.76) |  |  | 12(4.40) | 4(7.55) |  |  | 11(4.06) | 5(8.93) |  |  |
| **Employment** |  |  |  |  |  |  |  |  |  |  |  |  |  |  |
| Student | 98 (29.97) | 74(29.25) | 18(32.14) | 6(35.29) | 0.416 | 0.812 | 84(30.77) | 14(26.42) | 0.400 | 0.527 | 73(26.94) | 25(44.64) | 6.932 | **0.008** |
| Non-student | 229 (70.03) | 179(70.75) | 38(67.86) | 11(64.71) |  |  | 189(69.23) | 39(73.58) |  |  | 198(73.06) | 31(55.36) |  |  |
| **Monthly income (Chinese Yuan)** |  |  |  |  |  |  |  |  |  |  |  |  |  |  |
| <1500 | 59 (18.04) | 44(17.39) | 14(25.00) | 1(5.88) | — | 0.159* | 45(16.48) | 14(26.42) | 1.457 | **0.022** | 42(15.50) | 17(30.36) | 13.775 | **0.008** |
| 1500-3000 | 68 (20.80) | 53(20.95) | 7(12.50) | 8(47.06) |  |  | 63(23.08) | 5(9.43) |  |  | 57(21.03) | 11(19.64) |  |  |
| 3001-5000 | 96 (29.36) | 75(29.64) | 18(32.14) | 3(17.65) |  |  | 81(29.67) | 15(28.30) |  |  | 86(31.73) | 10(17.86) |  |  |
| 5001-8000 | 79 (24.16) | 60(23.72) | 14(25.00) | 5(29.41) |  |  | 61(22.34) | 18(33.96) |  |  | 69(25.46) | 10(17.86) |  |  |
| >8001 | 25 (7.65) | 21(8.30) | 3(5.36) | 0(0.00) |  |  | 23(8.42) | 1(1.89) |  |  | 17(6.27) | 8(14.29) |  |  |
| **sTable 2 Trajectory Group Characteristics of HIV Testing Patterns Among MSM** | | | | | | | | | | | | | | |
| **Variable** | **Allcount** | **HT** | | |  |  | **SBHT** | |  |  | **HIVST** | |  |  |
|  |  | Low (n=253) | Middle (n=56) | HigH (n=17) | *χ^2^/** | P | Low (n=273) | Middle (n=53) | *χ^2^/** | P | Low (n=271) | Middle (n=56) | *χ^2^/** | P |
| **Marital status** |  |  |  |  |  |  |  |  |  |  |  |  |  |  |
| Unmarried. | 293 (89.60) | 227(89.72) | 49(87.50) | 16(94.12) | — | 0.948* | 181(66.30) | 39(73.58) | 2.603 | 0.272 | 240(88.56) | 53(94.64) | — | 0.511* |
| Married. | 23 (7.03) | 17(6.72) | 5(8.93) | 1(5.88) |  |  | 71(26.01) | 13(24.53) |  |  | 21(7.75) | 2(3.57) |  |  |
| Divorced. | 11 (3.36) | 9(3.56) | 2(3.57) | 0(0.00) |  |  | 21(7.69) | 1(1.89) |  |  | 10(3.69) | 1(1.79) |  |  |
| **Sexual orientation** |  |  |  |  |  |  |  |  |  |  |  |  |  |  |
| Gay | 221 (67.58) | 173(68.38) | 35(62.50) | 12(70.59) | — | 0.778* | 181(66.30) | 39(73.58) | 2.603 | 0.272 | 180(66.42) | 41(73.21) | 1.479 | 0.477 |
| Bisexual | 84 (25.69) | 62(24.51) | 17(30.36) | 5(29.41) |  |  | 71(26.01) | 13(24.53) |  |  | 71(26.20) | 13(23.21) |  |  |
| Uncertain | 22 (6.73) | 18(7.11) | 4(7.14) | 0(0.00) |  |  | 21(7.69) | 1(1.89) |  |  | 20(7.38) | 2(3.57) |  |  |
| **Biological gender identity** |  |  |  |  |  |  |  |  |  |  |  |  |  |  |
| Yes | 286 (87.46) | 221(87.35) | 51(91.07) | 13(76.47) | 2.534 | 0.282 | 240(87.91) | 45(84.91) | 0.365 | 0.546 | 238(87.82) | 48(85.71) | 0.188 | 0.664 |
| No | 41 (12.54) | 32(12.65) | 5(8.93) | 4(23.53) |  |  | 33(12.09) | 8(15.09) |  |  | 33(12.18) | 8(14.29) |  |  |
| **Disclosure of sexual orientation** |  |  |  |  |  |  |  |  |  |  |  |  |  |  |
| Yes | 82 (25.08) | 60(23.72) | 17(30.36) | 5(29.41) | 1.247 | 0.536 | 69(25.27) | 13(24.53) | 0.013 | 0.909 | 66(24.35) | 16(28.57) | 0.439 | 0.507 |
| No | 245 (74.92) | 193(76.28) | 39(69.64) | 12(70.59) |  |  | 204(74.73) | 40(75.47) |  |  | 205(75.65) | 40(71.43) |  |  |
| **SBHT (past 6 months)** |  |  |  |  |  |  |  |  |  |  |  |  |  |  |
| Yes | 278 (85.02) | 60(23.72) | 17(30.36) | 5(29.41) | 1.247 | 0.536 | 232(84.98) | 45(84.91) | 0.000 | 0.989 | 233(85.98) | 45(80.36) | 1.151 | 0.283 |
| No | 49 (14.98) | 193(76.28) | 39(69.64) | 12(70.59) |  |  | 41(15.02) | 8(15.09) |  |  | 38(14.02) | 11(19.64) |  |  |
| **HIVST (past 6 months)** |  |  |  |  |  |  |  |  |  |  |  |  |  |  |
| Yes | 143 (43.59) |  |  |  |  |  |  |  |  |  |  |  |  |  |
| No | 185 (56.41) |  |  |  |  |  |  |  |  |  |  |  |  |  |
| **sTable 2 Trajectory Group Characteristics of HIV Testing Patterns Among MSM** | | | | | | | | | | | | | | |
| **Variable** | **Allcount** | **HT** | | |  |  | **SBHT** | |  |  | **HIVST** | |  |  |
|  |  | Low (n=253) | Middle (n=56) | HigH (n=17) | *χ^2^/** | P | Low (n=273) | Middle (n=53) | *χ^2^/** | P | Low (n=271) | Middle (n=56) | *χ^2^/** | P |
| **STI** |  |  |  |  |  |  |  |  |  |  |  |  |  |  |
| Yes | 30 (9.17) | 26(10.28) | 3(5.36) | 1(5.88) | 1.565 | 0.457 | 26(9.52) | 4(7.55) | 0.038 | 0.845 | 25(9.23) | 5(8.93) | 0.005 | 0.944 |
| No | 297 (90.83) | 227(89.72) | 53(94.64) | 16(94.12) |  |  | 247(90.48) | 49(92.45) |  |  | 246(90.77) | 51(91.07) |  |  |
| **Age of first sexual intercourse (year)** |  |  |  |  |  |  |  |  |  |  |  |  |  |  |
| <18 | 74 (22.63) | 59(23.32) | 12(21.43) | 3(17.65) | — | 0.873* | 61(22.34) | 13(24.53) | 0.163 | 0.922 | 58(21.40) | 16(28.57) | 2.628 | 0.269 |
| 18-30 | 236 (72.17) | 182(71.94) | 40(71.43) | 13(76.47) |  |  | 198(72.53) | 37(69.81) |  |  | 197(72.69) | 39(69.64) |  |  |
| >30 | 17 (5.20) | 12(4.74) | 4(7.14) | 1(5.88) |  |  | 14(5.13) | 3(5.66) |  |  | 16(5.90) | 1(1.79) |  |  |
| **Number of sexual partners (past 6 months)** |  |  |  |  |  |  |  |  |  |  |  |  |  |  |
| 0 | 182 (55.66) | 28(11.07) | 10(17.86) | 1(5.88) | — | 0.433* | 34(12.45) | 5(9.43) | 0.493 | 0.782 | 35(12.92) | 4(7.14) | 2.695 | 0.260 |
| 1-5 | 110 (33.64) | 201(79.45) | 41(73.21) | 13(76.47) |  |  | 213(78.02) | 42(79.25) |  |  | 212(78.23) | 44(78.57) |  |  |
| 6-30 | 35 (10.70) | 24(9.49) | 5(8.93) | 3(17.65) |  |  | 26(9.52) | 6(11.32) |  |  | 24(8.86) | 8(14.29) |  |  |
| **Sex role** |  |  |  |  |  |  |  |  |  |  |  |  |  |  |
| Inserter | 104 (31.80) | 74(29.25) | 25(44.64) | 4(23.53) | 7.328 | 0.120 | 81(29.67) | 22(41.51) | 3.039 | 0.219 | 192(70.85) | 38(67.86) | 4.957 | 0.084 |
| Inserted party | 100 (30.58) | 79(31.23) | 13(23.21) | 8(47.06) |  |  | 85(31.14) | 15(28.30) |  |  | 31(11.44) | 12(21.43) |  |  |
| Inserter/Inserted party | 123 (37.61) | 100(39.53) | 18(32.14) | 5(29.41) |  |  | 107(39.19) | 16(30.19) |  |  | 48(17.71) | 6(10.71) |  |  |
| **Positive sex partner in the past 6 months** |  |  |  |  |  |  |  |  |  |  |  |  |  |  |
| Yes | 230 (70.34) | 175(69.17) | 42(75.00) | 12(70.59) | — | 0.887* | 189(69.23) | 40(75.47) | 1.297 | 0.523 | 192(70.85) | 38(67.86) | 4.957 | 0.084 |
| No | 43 (13.15) | 34(13.44) | 6(10.71) | 3(17.65) |  |  | 36(13.19) | 7(13.21) |  |  | 31(11.44) | 12(21.43) |  |  |
| **sTable 2 Trajectory Group Characteristics of HIV Testing Patterns Among MSM** | | | | | | | | | | | | | | |
| **Variable** | **Allcount** | **HT** | | |  |  | **SBHT** | |  |  | **HIVST** | |  |  |
|  |  | Low (n=253) | Middle (n=56) | HigH (n=17) | *χ^2^/** | P | Low (n=273) | Middle (n=53) | *χ^2^/** | P | Low (n=271) | Middle (n=56) | *χ^2^/** | P |
| Uncertain | 54 (16.51) | 44(17.39) | 8(14.29) | 2(11.76) |  |  | 48(17.58) | 6(11.32) |  |  | 48(17.71) | 6(10.71) |  |  |
| **Experienced condom breakage (past 6 months)** |  |  |  |  |  |  |  |  |  |  |  |  |  |  |
| Yes | 237 (72.48) | 144(56.92) | 30(53.57) | 7(41.18) | 1.702 | 0.427 | 154(56.41) | 27(50.94) | 0.537 | 0.464 | 157(57.93) | 25(44.64) | 3.322 | 0.068 |
| No | 90 (27.52) | 109(43.08) | 26(46.43) | 10(58.82) |  |  | 119(43.59) | 26(49.06) |  |  | 114(42.07) | 31(55.36) |  |  |
| **Drug use** |  |  |  |  |  |  |  |  |  |  |  |  |  |  |
| Yes | 82 (25.08) | 60(23.72) | 17(30.36) | 5(29.41) | 1.247 | 0.536 | 64(23.44) | 18(33.96) | 2.609 | 0.106 | 60(22.14) | 22(39.29) | 7.261 | **0.007** |
| No | 245 (74.92) | 193(76.28) | 39(69.64) | 12(70.59) |  |  | 209(76.56) | 35(66.04) |  |  | 211(77.86) | 34(60.71) |  |  |
| HT: HIV testing. SBHT: HIV facility-based testing. HIVST: HIV facility-based testing. | | | | | | | | | | | | | | |
