# Supplementary material for: Exploring the relationship between HIV Testing frequency and sexual behavior, prevention prophylaxis use among MSM in Southern China
Source: PLoS One. 2026 Sep 29;21(9):e0358578. doi: 10.1371/journal.pone.0358578 (PMC13623135; doi:10.1371/journal.pone.0358578)
Supplement: Supplementary file 3 [file pone.0358578.s003.docx]

## **sTable 3. Descriptive changes in HT, HIVST, SBHT, PEP/PEP, unprotected sexual behavior from baseline to 12-month follow-up**

| Indicator | T0 | T1 | T2 | T3 | T4 |
| --- | --- | --- | --- | --- | --- |
| **HIV testing frequency (tests per 3 months), Mean ± SD** |  |  |  |  |  |
| Overall HIV testing | 3.35 ± 2.43 | 1.23 ± 1.19 | 1.27 ± 1.14 | 1.22 ± 1.10 | 1.16 ± 1.18 |
| HIV self-testing (HIVST) | 1.10 ± 1.78 | 0.82 ± 1.16 | 0.66 ± 0.98 | 0.54 ± 0.92 | 0.58 ± 1.09 |
| Facility-based testing (SBHT) | 2.25 ± 1.93 | 0.38 ± 0.62 | 0.55 ± 0.74 | 0.62 ± 0.82 | 0.52 ± 0.72 |
| **Tested at least once, n (%)** |  |  |  |  |  |
| Overall HIV testing | 327(100) | 214(65.44) | 224(68.50) | 223(68.20) | 207(63.30) |
| **HIV exposure prophylaxis use, n (%)** |  |  |  |  |  |
| PrEP | 19(5.81) | 13(3.98) | 13(3.98) | 17(5.20) | 18(5.50) |
| PEP | 54(16.51) | 10(3.06) | 8(2.45) | 6(1.83) | 9(2.75) |
| **Unprotected sexual behavior** |  |  |  |  |  |
| Mean ± SD (acts per 3 months) | 6.58 ± 16.37 | 1.26 ± 3.04 | 1.36 ± 3.04 | 0.99 ± 2.56 | 1.31 ± 2.88 |
| Occurred, n (%) | 182 (55.66) | 81(24.77) | 87(26.60) | 71(21.71) | 84(25.69) |

*Note: T0: baseline; T1–T4: 3-, 6-, 9-, and 12-month follow-up visits. PrEP: pre-exposure prophylaxis; PEP: post-exposure prophylaxis; HIVST: HIV self-testing; SBHT: site-based HIV testing. Values are based on observed data; no formal statistical testing was performed.*

*All participants (100%) had undergone HIV testing at least once in the past 12 months per the eligibility criteria (participants were required to be HIV-negative at enrollment, which necessitated prior or baseline testing).*

*For HIV testing frequency, baseline reflects the past 12 months (tests per year), whereas T1–T4 reflect the past 3 months (tests per 3 months).*

*For PrEP and PEP use, baseline reflects “ever used” (lifetime use) at enrollment, whereas T1–T4 reflect use within the past 3 months at each follow-up visit.*

*For unprotected sexual behavior, baseline reflects the past 6 months, whereas T1–T4 reflect the past 3 months.*

*During the 12-month follow-up period, a total of 22 new STI cases were diagnosed.*
