# Supplementary material for: Exploring the relationship between HIV Testing frequency and sexual behavior, prevention prophylaxis use among MSM in Southern China
Source: PLoS One. 2026 Sep 29;21(9):e0358578. doi: 10.1371/journal.pone.0358578 (PMC13623135; doi:10.1371/journal.pone.0358578)
Supplement: Supplementary file 4 [file pone.0358578.s005.docx]

**Response to Reviewers**

**Manuscript ID:** PONE-D-25-58406

**Title:** Exploring the Relationship Between HIV Testing Frequency and Sexual Behavior, Prevention Prophylaxis Use Among MSM in Southern China

**Journal:** PLOS ONE

Dear Dr. Bloom and Reviewers,

Thank you for the opportunity to revise and resubmit our manuscript to PLOS ONE. We sincerely appreciate the thoughtful and constructive comments from both reviewers, which have significantly improved the quality of our work. We have carefully addressed all concerns point-by-point as detailed below. All changes in the manuscript have been highlighted using track changes.

**Journal Requirements**

**Response to Requirement 1 (Formatting):** We have reformatted the manuscript according to the PLOS ONE style templates. The title page, author affiliations, main body, and references now follow the journal's formatting guidelines. All files have been named as requested.

**Response to Requirement 2 (Funding Statement):** We have amended the Funding Statement as follows:

“This study was supported by the Outstanding Youth Fund of the Natural Science Foundation of Guangxi in China (Grant No. 2023GXNSFFA026007); the Natural Science Foundation of Guangxi in China (Grant No. 2023GXNSFAA026287); and the Science and Technology Project for Disease Control and Prevention of Guangxi in China (Grant No. GXJKKJ2025YB00). There was no additional external funding received for this study.”

The amended statement has been included in the cover letter. All funding-related text has been removed from the manuscript.

**Response to Requirement 3 (Funding in Manuscript):** All funding-related content has been removed from the manuscript. Funding information now appears only in the online submission form.

**Response to Requirement 4 (Data Availability Statement):** We have updated the Data Availability Statement. The raw data supporting the findings of this study are available from the Institutional Review Board at Guangxi Medical University upon reasonable request. Due to participant privacy concerns (including sensitive information about sexual behavior and HIV status), data cannot be made publicly available. Requests for data access may be directed to the corresponding author or to the Institutional Review Board at Guangxi Medical University (irb@gxmu.edu.cn).

**Response to Requirement 5 (Ethics Statement):** The ethics statement has been moved to the Methods section under a dedicated "Ethics Statement" subsection. It has been removed from all other sections.

**Responses to Reviewer #1**

***Reviewer #1, Comment 1:** Although this study uses a longitudinal cohort with repeated quarterly measures, the analysis primarily focuses on trajectory group membership and between-group differences. It is unclear how outcomes (e.g., HIV testing frequency, PrEP/PEP use, sexual behaviors) changed from baseline to follow-up within individuals. The authors should clarify whether and how within-person changes over time were assessed.

**Response:** We thank the reviewer for this valuable methodological suggestion. We fully agree that assessing within-person changes over time is important in longitudinal studies. However, the main objective of our study was to identify distinct longitudinal trajectories of HIV testing frequency and to examine how these trajectory groups differ in terms of prevention uptake and sexual risk behaviors—rather than to model individual-level change over time per se. We believe the trajectory-based approach (GBTM) is appropriate for this research question, as it captures heterogeneity in testing patterns and allows group-level comparisons using Cox regression, which directly addresses our primary aim.

That said, we acknowledge that we did not explicitly report within-person changes from baseline to end of follow-up. To address this concern without introducing additional statistical models that fall outside our pre-specified analysis plan, we have:

1.Added a **limitation statement** in the Limitation section acknowledging that we did not apply mixed-effects or GEE models to formally test within-person change, while noting that GBTM inherently captures individual-level longitudinal patterns by grouping participants who follow similar developmental trajectories.

2.Added descriptive comparisons of key outcomes between baseline and follow-up in the Discussion to provide readers with a general sense of observed changes.

(See Limitation in the revised manuscript, lines 27-35, page 10)

***Reviewer #1, Comment 2:** *The manuscript reports recruitment between September 2021 and February 2022, with follow-up completed by February 2023, but elsewhere states that follow-up lasted “until March 2022.” These statements are inconsistent.*

**Response:** We apologize for this inconsistency. We have clarified the study timeline as follows:

**Baseline recruitment:** September 1, 2021 to February 28, 2022

**Follow-up schedule:** 3, 6, 9, and 12 months after baseline

**Final follow-up completion:** February 28, 2023

**Maximum follow-up duration:** 12 months for all participants

The inconsistent statement has been corrected.

(See Methods in the revised manuscript, lines 10-14, page 4)

***Reviewer #1, Comment 3:** *The study period overlaps with COVID-19 control measures in China, which may have affected access to HIV testing and PrEP services.*

**Response:** We agree with the reviewer. We have added a discussion of this limitation in the Limitation section, noting that while study sites did not experience strict lockdowns during recruitment, local mobility restrictions and temporary clinic closures may have affected access to services. We have explicitly stated that readers should interpret the findings with consideration of this unique pandemic context.

(See Limitations in the revised manuscript, lines 8-14, page 10)

***Reviewer #1, Comment 4:** The definitions of “low,” “middle,” and “high” HIV testing frequency are inconsistent between the main text and figure legend. The rationale for the chosen cut-points should be justified.

**Response:** We thank the reviewer for catching this inconsistency. We have:

1. Harmonized the definitions across the manuscript based on recommendations from multiple international guidelines: the WHO (2019) recommends annual testing for sexually active MSM and 3–6 monthly testing for those at increased risk; the US CDC (2025) recommends annual screening for MSM, with more frequent testing (every 3–6 months) for high-risk individuals; and the IAS-USA (2024) advises 6-monthly HIV testing for PrEP users and 3-monthly testing for frequently exposed individuals.
2. Final definitions: Low (<1 test/3 months), Medium (1–2 tests/3 months), and High (≥3 tests/3 months).
3. Added justification in the Methods section and updated the Figure 1 legend accordingly.

(See Methods in the revised manuscript, lines 3-10, page 5, and Fig 1 legend)

***Reviewer #1, Comment 5:** The sample is relatively young and includes a high proportion of students, which may not fully represent the broader MSM population in China.

**Response:** We agree with the reviewer. We have:

1. Added comparisons with other Chinese MSM cohorts in the Limitations section: a cohort study in Zhuhai reported that MSM aged 18–24 accounted for 23.18%, with students comprising only 6.62%; a 28-province study (N= 2,618 ) reported 26.90% aged <25 years and 18.80% students; and a Northwest China study reported 16.3% aged 18–25 years with <8.1% students.
2. Explicitly stated that these comparisons suggest our sample is significantly younger and more student-dominated than many other Chinese MSM cohorts, limiting generalizability to older MSM and non-student populations.
3. Called for future studies with more diverse age and occupational groups to validate our findings.

(See Limitation in the revised manuscript, lines 15-24, page 10)

***Reviewer #1, Comment 6:** The terms “pretreatment” and “post-treatment” visits are unclear in the context of this cohort analysis and may imply an intervention effect.

Response: These terms have been replaced with "baseline" and "follow-up" throughout the manuscript.

(See Methods in the revised manuscript, lines 24-25, page 4)

***Reviewer #1, Comment 7: ***The authors may consider discussing digital tools in improving HIV testing, such as doi:10.1001/jamanetworkopen.2025.44821*

**Response:** We thank the reviewer for this suggestion. We have added a brief discussion on digital tools in improving HIV testing among MSM in the Discussion section, citing the recommended reference (doi:10.1001/jamanetworkopen.2025.44821). We note that integrating such digital approaches into HIV prevention programs in southern China may complement facility-based testing and help address barriers such as stigma and privacy concerns.

(See Discussion in the revised manuscript, lines 1-6, page 9, and Reference 36)

***Reviewer #1, Comment 8:** *Table 1 appears to contain potential internal inconsistencies (e.g., reporting zero sexual partners while also reporting a high proportion of HIV-positive partners).*

We thank the reviewer for carefully identifying this issue. We sincerely apologize for this error. Upon re-examination, we found that the inconsistencies in Table 1 were caused by data transcription errors during the preparation of the manuscript. Specifically:

The variable "condomless anal sex with men (past 6 months)" was incorrectly placed: the correct values should be Yes: 182 (55.66%), No: 145 (44.34%). These data were previously assigned to the wrong row.

The variable "positive sex partner in the past 6 months" was also misclassified. The correct values are Yes: 43 (13.15%), No: 230 (70.34%), Uncertain: 54 (16.51%).

We have now carefully verified all data entries in Table 1 against the original dataset and corrected the erroneous row placements. The revised Table 1 has been updated accordingly, and all corrections have been highlighted in the revised manuscript.

To prevent such errors in the future, we have implemented a double-check procedure: all tables will now be independently verified by a second author against the original data before submission.

We sincerely apologize for these oversights and appreciate the reviewer's diligence in catching these issues.

(See Table 1)

**Responses to Reviewer #2**

***Reviewer #2 (General Comment):** Very nicely laid out and written manuscript. Would revise to focus on 'Use of PrEP' among trajectories as primary objective and STI as secondary. The only technical concern is that some of the subgroups trajectories fall below 5% of the study population which may indicate that alternative grouping strategies need to be considered. Conclusion should also reconcile differences between PrEP and PEP uptake.

**Response:** Thank you for the positive feedback. We have:

1. Refocused the manuscript to present PrEP/PEP use as the primary outcome and STI as secondary, with adjustments made to the Abstract, Introduction, Methods, and Results sections.
2. Addressed the <5% subgroup issue by adding an explanation that although the 4.8% and 4.91% proportions are slightly below the pre-specified 5% threshold, sensitivity analyses with alternative model specifications (2-group and 4-group solutions) confirmed the presence of these distinct trajectory groups. The original 3-group solution was retained because it provided the best fit to the data (lowest BIC) and clinical interpretability.
3. Revised the Conclusion to separately discuss PrEP and PEP: PrEP use was more strongly associated with HIV self-testing, while PEP use was more closely linked to facility-based testing.

(See Introduction in the revised manuscript lines 42-43, page 3, Results lines 3-13, page 8, and Conclusion lines 39-43, page 10)

**Reviewer #2 (Abstract Line #17):** "men" not "man"

**Response:** Corrected. "man who have sex with man" has been changed to "men who have sex with men (MSM)" throughout the manuscript.

**Reviewer #2 (Introduction):** "INTRODUCTION" not "INTRUDITION"

**Response:** Corrected.

***Reviewer #2 (Introduction Lines 18-19):** I believe that authors wanted to state that "retest" rates were less than 20%*

**Response:** We have clarified this statement: "An Australian study indicated that HIV retest rates among MSM were below 40% after one year and below 20% after six months."

**Reviewer #2 (Introduction Line 42 & Methods Line 26):** Aim in examining STI incidence is not consistent with abstract.

**Response:** We have revised these sentences to clarify that PrEP/PEP use is the primary objective and STI incidence is secondary.

(See Introduction in the revised manuscript lines 42-43, page 3, Results lines 3-13, page 8, and Conclusion lines 39-43, page 10)

**Reviewer #2 (Statistics Line 8):** Please include actual and minimum allowable posterior assignment probability.

**Response:** We have added: "the minimum acceptable posterior assignment probability for trajectory group membership was set at ≥0.8."

(See Methods in the revised manuscript, lines 17-18, page 5)

**Reviewer #2 (Results Line 4 and 11):** Please explain why high use of 4.8% and 4.91% were allowed when 5% was minimum.

**Response:** We have added an explanation in the Statistics section: "Although our pre-specified minimum group size was 5%, the 4.8% and 4.91% proportions represent slight deviations due to model optimization based on BIC. We conducted sensitivity analyses with alternative model specifications (2-group and 4-group solutions), which confirmed the presence of distinct trajectory groups. The original 3-group solution was retained because it provided the best fit to the data (lowest BIC) and clinical interpretability."

(See Methods in the revised manuscript, lines 18-23, page 5)

**Reviewer #2:** Would reorder Incidence of STI and Use of HIV Exposure Prophylaxis actions.

**Response:** The Results section has been reordered as suggested: "Use of HIV exposure prophylaxis (PrEP/PEP)" now appears before "Incidence and risk of STI."

(See Results in the revised manuscript, lines 7-13, page 8)

**Reviewer #2 (Discussion and Conclusion):** Statistics on condom use should be highlighted earlier; age and PEP association should be discussed.

**Response:** We have:

1. Added condom use descriptive statistics in the baseline characteristics section of the Results.
2. Added a discussion of potential barriers to linking self-testing to PEP for younger MSM: lack of awareness about PEP availability, perceived low risk, cost concerns, and fear of disclosure.

(See Results in the revised manuscript, lines 12-13, page 6, and Discussion lines 1-6, page 9)

**Conclusion**

We sincerely thank the editor and both reviewers for their time, expertise, and constructive feedback, which have been invaluable in strengthening our manuscript. We have made every effort to address all comments thoroughly and believe that the revised version is substantially improved.

We acknowledge that some of the methodological concerns raised—particularly regarding within-person change analyses—represent important avenues for future research. We are fully open to making additional revisions should the editor or reviewers feel that further improvements are needed. Please do not hesitate to let us know if any aspect of our response or revision requires additional clarification or adjustment; we would be happy to address it promptly.

We appreciate your continued consideration of our work and look forward to your further guidance.

Sincerely,

Ning Chuanyi, MD, PhD

Corresponding author

On behalf of all authors
