# Supplementary material for: Exploring the relationship between HIV Testing frequency and sexual behavior, prevention prophylaxis use among MSM in Southern China
Source: PLoS One. 2026 Sep 29;21(9):e0358578. doi: 10.1371/journal.pone.0358578 (PMC13623135; doi:10.1371/journal.pone.0358578)
Supplement: Supplementary file 5 [file pone.0358578.s006.docx]

**Manuscript ID:** PONE-D-25-58406R1

**Title:** Exploring the Relationship Between HIV Testing Frequency and Sexual Behavior, Prevention Prophylaxis Use Among MSM in Southern China

**Journal:** PLOS ONE

Dear Dr. Nguyen and Reviewers,

Thank you very much for giving us the opportunity to revise and resubmit our manuscript to PLOS ONE. We are sincerely grateful to the reviewers for taking the time to carefully evaluate our work. The constructive comments have been invaluable in strengthening our manuscript. We have addressed all concerns point-by-point as detailed below. All changes in the manuscript have been highlighted using track changes.

**Responses to Reviewer #3**

***Reviewer #3, Comment 1:** The definitions of low-, middle-, and high-frequency HIV testing remain inconsistent between the Methods section and Figure 1. In the Methods, the categories are defined as low (<1 test/3 months), middle (1–2 tests/3 months), and high (≥3 tests/3 months). However, the Figure 1 legend still defines the middle group as “1 time every 3 months” and the high group as “≥2 times every 3 months.”

**Response:** We thank the reviewer for this careful observation. We have now thoroughly harmonized the definitions across the entire manuscript. The final definitions are: Low frequency (<1 test per 3 months), Medium frequency (1–2 tests per 3 months), and High frequency (≥3 tests per 3 months). These definitions have been consistently applied in the Methods section, Results section, Fig 1 (main text and legend). All corresponding text and figure legends have been revised and highlighted in the updated manuscript.

(See Results section: "Definition of Trajectory Groups"; Fig 1 legend and Fig 1)

***Reviewer #3, Comment 2:** In the response letter, the authors state that descriptive comparisons between baseline and follow-up outcomes were added. However, I could not clearly identify these comparisons in the revised manuscript.

**Response:** We thank the reviewer for this comment. In the previous revision, we added descriptive comparisons between baseline and follow-up outcomes in the Discussion section, specifically within the paragraph on descriptive trends. To present these data more clearly, we have also provided the full descriptive comparison table as Supplementary Table S3. This table includes key indicators such as HIV testing frequency (overall, HIVST, and SBHT), PrEP/PEP use, unprotected sexual behavior, and STI cases from baseline to each follow-up time point.

We have noted in both the table and the Discussion that baseline recall periods differ from follow-up: HIV testing reflects the past 12 months at baseline versus the past 3 months at follow-up; unprotected sexual behavior reflects the past 6 months at baseline versus the past 3 months at follow-up; and PrEP/PEP use reflects lifetime use at baseline versus past-3-month use at follow-up. These descriptive data are intended to provide readers with an overview of raw trends and no formal statistical testing was performed.

(See Discussion section, lines 24-34, page 8; Supplementary sTable3)

***Reviewer #3, Comment 3:** The response letter indicates that discussion was added regarding barriers to PEP uptake among younger MSM. I was unable to locate this discussion in the revised manuscript.

**Response:** We thank the reviewer for pointing this out. We apologize for this omission. We have now added a discussion of barriers to PEP uptake among younger MSM in the Discussion section. We note that there is a gap between frequent HIV self-testing among younger MSM and low PEP uptake. Potential barriers include limited awareness of PEP access, perceived low risk despite frequent testing, out-of-pocket cost concerns, and fear of stigma when seeking PEP at clinics. These findings suggest that integrating PEP education into self-testing platforms and establishing rapid referral pathways may help bridge this gap, particularly for younger MSM.

(See Discussion section, lines 11-23, page 10)

***Reviewer #3, Comment 4:** The manuscript states that sensitivity analyses using alternative trajectory models confirmed the selected model and refers readers to Supplementary Table S1. Please ensure that this supplementary table is included in the submission.

**Response:** We confirm that Supplementary Table S1 has been included in the revised submission. This table contains the model fit assessment indicators (BIC, AIC, and posterior assignment probabilities) for all trajectory models. The table is referenced in the Methods section (Statistical Analysis paragraph) and has been uploaded as part of the Supporting Information files.

(See Supplementary sTable1)

***Reviewer #3** (Minor inconsistencies and typographical errors):

**Response:** We have carefully corrected all editorial issues identified by the reviewer:

Fig 1 legend: Corrected the erroneous description from "(C) HIVST: HIV facility-based testing" to "(C) HIVST: HIV self-testing."

Duplicate words: Corrected "Use of HIV exposure prophylaxis prophylaxis" to "Use of HIV exposure prophylaxis" in the Results section heading.

Duplicate punctuation: Removed all instances of double periods ("..") throughout the manuscript.

Spacing and formatting: Corrected minor spacing and formatting inconsistencies throughout the manuscript.

Grammatical error: Rewrote "The World Health Organization (WHO) has HIVST serves as a strategy..." to "The World Health Organization (WHO) recommends HIVST as a strategy to enhance the accessibility of HT for individuals at elevated risk."

Data Availability Statement: Replaced the inappropriate statement with the correct Data Availability Statement.

***Reviewer #3** (Consistency between the response letter and manuscript):

**Response:** We sincerely thank the reviewer for this important reminder. We have carefully cross-checked every statement in the response letter against the actual revised manuscript. All promised revisions have been implemented and highlighted in the revised manuscript with track changes.

**Journal Requirements**

**Response to Data Availability Statement:** We have updated the Data Availability Statement to comply with PLOS ONE's policy. The revised statement reads:

"The raw data supporting the findings of this study are not publicly available due to the sensitive nature of the information collected (including sexual behavior and HIV status), which could compromise participant privacy. Data requests may be directed to the Institutional Review Board at Guangxi Medical University (gxmuyfyll@163.com), which will review requests for data access in accordance with the ethical approval granted for this study."

(See Methods section, lines 39-43, page 5)

Conclusion

We sincerely thank the editor and reviewers for their valuable time and expert guidance in improving our manuscript. Each comment has been deeply insightful and has contributed significantly to strengthening our work. We have made every effort to address all comments thoroughly and believe that the revised version is substantially improved in both academic rigor and completeness of presentation.

We fully recognize that scholarly writing is an ongoing process. Should the editor or reviewers feel that further improvements are needed, we remain entirely open to making additional revisions. Please do not hesitate to let us know if any aspect of our response or revision requires further clarification or adjustment; we would be happy to address it promptly.

We sincerely appreciate your continued consideration of our work and look forward to your final decision.

Sincerely,

Chuanyi Ning, MD, PhD

Corresponding author

On behalf of all authors
