# Supplementary material for: Exploring the relationship between HIV Testing frequency and sexual behavior, prevention prophylaxis use among MSM in Southern China
Source: PLoS One. 2026 Sep 29;21(9):e0358578. doi: 10.1371/journal.pone.0358578 (PMC13623135; doi:10.1371/journal.pone.0358578)
Supplement: Peer review history

# Peer review: Decision Letter 0

**DOI:** 10.1371/journal.pone.0358578.r001

**Authors:** Ilse Bloom

10 Apr 2026

Dear Dr. Ning,

The manuscript has been evaluated by two reviewers, and their comments are available below.

Could you please carefully revise the manuscript to address all comments raised?

Please submit your revised manuscript by May 24 2026 11:59PM. If you will need more time than this to complete your revisions, please reply to this message or contact the journal office at plosone@plos.org. When you're ready to submit your revision, log on to https://www.editorialmanager.com/pone/ and select the 'Submissions Needing Revision' folder to locate your manuscript file.

- A letter that responds to each point raised by the academic editor and reviewer(s). You should upload this letter as a separate file labeled 'Response to Reviewers'.
- A marked-up copy of your manuscript that highlights changes made to the original version. You should upload this as a separate file labeled 'Revised Manuscript with Track Changes'.
- An unmarked version of your revised paper without tracked changes. You should upload this as a separate file labeled 'Manuscript'.

If you would like to make changes to your financial disclosure, please include your updated statement in your cover letter. Guidelines for resubmitting your figure files are available below the reviewer comments at the end of this letter.

If applicable, we recommend that you deposit your laboratory protocols in protocols.io to enhance the reproducibility of your results. Protocols.io assigns your protocol its own identifier (DOI) so that it can be cited independently in the future. For instructions see: https://journals.plos.org/plosone/s/submission-guidelines#loc-laboratory-protocols. Additionally, PLOS ONE offers an option for publishing peer-reviewed Lab Protocol articles, which describe protocols hosted on protocols.io. Read more information on sharing protocols at https://plos.org/protocols?utm_medium=editorial-email&utm_source=authorletters&utm_campaign=protocols.

As the corresponding author, your ORCID iD is verified in the submission system and will appear in the published article. PLOS supports the use of ORCID, and we encourage all coauthors to register for an ORCID iD and use it as well. Please encourage your coauthors to verify their ORCID iD within the submission system before final acceptance, as unverified ORCID iDs will not appear in the published article. *Only*  the individual author can complete the verification step; PLOS staff *cannot*  verify ORCID iDs on behalf of authors.

We look forward to receiving your revised manuscript.

Kind regards,

Ilse Bloom

Staff Editor

PLOS One

Journal Requirements:

When submitting your revision, we need you to address these additional requirements.

1. Please ensure that your manuscript meets PLOS ONE's style requirements, including those for file naming. The PLOS ONE style templates can be found at

https://journals.plos.org/plosone/s/file?id=wjVg/PLOSOne_formatting_sample_main_body.pdf and https://journals.plos.org/plosone/s/file?id=ba62/PLOSOne_formatting_sample_title_authors_affiliations.pdf

2. Thank you for stating in your Funding Statement:

[This study received support from the Outstanding Youth Fund of The Natural Science Foundation of Guangxi in China (Grant No. 2023GXNSFFA026007); The Natural Science Foundation of Guangxi in China (Grant No. 2023GXNSFAA026287); The Science and Technology Project for Disease Control and Prevention of Guangxi in China (Grant No. GXJKKJ2025YB00).].

Please provide an amended statement that declares *all* the funding or sources of support (whether external or internal to your organization) received during this study, as detailed online in our guide for authors at http://journals.plos.org/plosone/s/submit-now.  Please also include the statement “There was no additional external funding received for this study.” in your updated Funding Statement.

Please include your amended Funding Statement within your cover letter. We will change the online submission form on your behalf.

3. Please remove any funding-related text from the manuscript. Funding information should not appear in any section or other areas of your manuscript. We will only publish funding information present in the Funding Statement section of the online submission form.

4. Please note that your Data Availability Statement is currently missing the repository name and/or the DOI/accession number of each dataset OR a direct link to access each database. If your manuscript is accepted for publication, you will be asked to provide these details on a very short timeline. We therefore suggest that you provide this information now, though we will not hold up the peer review process if you are unable.

5. Your ethics statement should only appear in the Methods section of your manuscript. If your ethics statement is written in any section besides the Methods, please delete it from any other section.

If the reviewer comments include a recommendation to cite specific previously published works, please review and evaluate these publications to determine whether they are relevant and should be cited. There is no requirement to cite these works unless the editor has indicated otherwise.

[Note: HTML markup is below. Please do not edit.]

Reviewers' comments:

Reviewer's Responses to Questions

**Comments to the Author**

1. Is the manuscript technically sound, and do the data support the conclusions?

Reviewer #1: No

Reviewer #2: Partly

**********

2. Has the statistical analysis been performed appropriately and rigorously?

Reviewer #1: No

Reviewer #2: Yes

**********

3. Have the authors made all data underlying the findings in their manuscript fully available?

The PLOS Data policy

Reviewer #1: Yes

Reviewer #2: Yes

**********

4. Is the manuscript presented in an intelligible fashion and written in standard English?

Reviewer #1: Yes

Reviewer #2: Yes

**********

Reviewer #1: 1. Although this study uses a longitudinal cohort with repeated quarterly measures, the analysis primarily focuses on trajectory group membership and between-group differences. It is unclear how outcomes (e.g., HIV testing frequency, PrEP/PEP use, sexual behaviors) changed from baseline to follow-up within individuals. The authors should clarify whether and how within-person changes over time were assessed (e.g., baseline vs endline, time trends using mixed-effects or GEE models).

2. The manuscript reports recruitment between September 2021 and February 2022, with follow-up completed by February 2023, but elsewhere states that follow-up lasted “until March 2022.” These statements are inconsistent. The authors should clearly define: (a) what constitutes baseline, (b) the exact follow-up schedule (e.g., 3, 6, 9, 12 months), and (c) the maximum follow-up duration used in analyses.

3. The study period overlaps with COVID-19 control measures in China, which may have affected access to HIV testing and PrEP services among MSM. Although the authors state that study sites did not experience strict lockdowns during recruitment, variation in service availability over calendar time may still bias outcomes. The authors should more explicitly discuss this issue and, if possible, consider sensitivity analyses adjusting for calendar period.

4. The definitions of “low,” “middle,” and “high” HIV testing frequency are inconsistent between the main text and figure legend. These categories should be harmonized across the manuscript. In addition, the rationale for the chosen cut-points should be justified (e.g., based on guidelines, prior literature, or empirical distribution), and discussed in the main text.

5. The sample is relatively young and includes a high proportion of students, which may not fully represent the broader MSM population in China. The authors should compare baseline characteristics with other Chinese MSM cohorts and discuss implications for generalizability.

6. The terms “pretreatment” and “post-treatment” visits are unclear in the context of this cohort analysis and may imply an intervention effect. Please clarify what “treatment” refers to and whether trial arm assignment was accounted for in the analyses.

7. The authors may consider discussing digital tools in improving HIV testing, such as doi:10.1001/jamanetworkopen.2025.44821

8. Table 1 appears to contain potential internal inconsistencies (e.g., reporting zero sexual partners while also reporting a high proportion of HIV-positive partners). The authors should verify variable definitions and data coding.

Reviewer #2: Very nicely laid out and written manuscript. Would revise to focus on 'Use of PrEP" among trajectories as primary objective and STI as secondary. The only technical concern is that some of the subgroups trajectories fall below 5% of the study population which may indicate that alternative grouping strategies (different cut-offs or less groups) need to be considered. Conclusion should also reconcile differences between PrEP and PEP uptake.

A few specific comments below:

Abstract

Line #17 "men" not "man"

Manuscript

Introduction

- "INTRODUCTION" not INTRUDITION

- Lines 18 and 19, I believe that authors wanted to state that "retest" rates were less than 20%

- Line 42, aim in examining STI incidence is not consistent with abstract. STI is secondary outcome for this paper. Please revise if that was intention of this manuscript.

Methods

- Line 26, aim in examining STI incidence is not consistent with abstract. Use of PreP is. STI is secondary outcome for this paper. Please revise if that was intention of this manuscript.

Statistics

-Line 8, please include actual and minimum allowable posterior assignment probability (e.g. >0.8 was considered acceptable)

Results

- Line 4 and 11, please explain why high use of 4.8% and 4.91% of study population, respectively were allowed, when 5% was minimum in Methods

- Would reorder Incidence of STI and Use of HIV Exposure Prophylaxis actions

Discussion and Conclusion

I did not see statistics on condom use highlighted early in manuscript. Descriptive data should be laid out prior to discussing in conclusion. Also, per appendix, age is correlated with self-testing but middle trajectories not correlated with PEP, please include in your discussion on how this gap can be potentially addressed in the local context. What are potential bariers to linking ST to PEP for this age group which uses high rates of self-testing? Can this be explained?

**********

what does this mean?). If published, this will include your full peer review and any attached files.

If you choose “no”, your identity will remain anonymous but your review may still be made public.

**Do you want your identity to be public for this peer review?**  For information about this choice, including consent withdrawal, please see our Privacy Policy

Reviewer #1: No

Reviewer #2: No

**********

[NOTE: If reviewer comments were submitted as an attachment file, they will be attached to this email and accessible via the submission site. Please log into your account, locate the manuscript record, and check for the action link "View Attachments". If this link does not appear, there are no attachment files.]

To ensure your figures meet our technical requirements, please review our figure guidelines: https://journals.plos.org/plosone/s/figures

You may also use PLOS’s free figure tool, NAAS, to help you prepare publication quality figures: https://journals.plos.org/plosone/s/figures#loc-tools-for-figure-preparation.

NAAS will assess whether your figures meet our technical requirements by comparing each figure against our figure specifications.

# Author comment: Author response to Decision Letter 1

**DOI:** 10.1371/journal.pone.0358578.r002

22 Jun 2026

Response to Reviewers

Manuscript ID: PONE-D-25-58406

Title: Exploring the Relationship Between HIV Testing Frequency and Sexual Behavior, Prevention Prophylaxis Use Among MSM in Southern China

Journal: PLOS ONE

Dear Dr. Bloom and Reviewers,

Thank you for the opportunity to revise and resubmit our manuscript to PLOS ONE. We sincerely appreciate the thoughtful and constructive comments from both reviewers, which have significantly improved the quality of our work. We have carefully addressed all concerns point-by-point as detailed below. All changes in the manuscript have been highlighted using track changes.

Journal Requirements

Response to Requirement 1 (Formatting): We have reformatted the manuscript according to the PLOS ONE style templates. The title page, author affiliations, main body, and references now follow the journal's formatting guidelines. All files have been named as requested.

Response to Requirement 2 (Funding Statement): We have amended the Funding Statement as follows:

“This study was supported by the Outstanding Youth Fund of the Natural Science Foundation of Guangxi in China (Grant No. 2023GXNSFFA026007); the Natural Science Foundation of Guangxi in China (Grant No. 2023GXNSFAA026287); and the Science and Technology Project for Disease Control and Prevention of Guangxi in China (Grant No. GXJKKJ2025YB00). There was no additional external funding received for this study.”

The amended statement has been included in the cover letter. All funding-related text has been removed from the manuscript.

Response to Requirement 3 (Funding in Manuscript): All funding-related content has been removed from the manuscript. Funding information now appears only in the online submission form.

Response to Requirement 4 (Data Availability Statement): We have updated the Data Availability Statement. The raw data supporting the findings of this study are available from the Institutional Review Board at Guangxi Medical University upon reasonable request. Due to participant privacy concerns (including sensitive information about sexual behavior and HIV status), data cannot be made publicly available. Requests for data access may be directed to the corresponding author or to the Institutional Review Board at Guangxi Medical University (irb@gxmu.edu.cn).

Response to Requirement 5 (Ethics Statement): The ethics statement has been moved to the Methods section under a dedicated "Ethics Statement" subsection. It has been removed from all other sections.

Responses to Reviewer #1

*Reviewer #1, Comment 1: Although this study uses a longitudinal cohort with repeated quarterly measures, the analysis primarily focuses on trajectory group membership and between-group differences. It is unclear how outcomes (e.g., HIV testing frequency, PrEP/PEP use, sexual behaviors) changed from baseline to follow-up within individuals. The authors should clarify whether and how within-person changes over time were assessed.

Response: We thank the reviewer for this valuable methodological suggestion. We fully agree that assessing within-person changes over time is important in longitudinal studies. However, the main objective of our study was to identify distinct longitudinal trajectories of HIV testing frequency and to examine how these trajectory groups differ in terms of prevention uptake and sexual risk behaviors—rather than to model individual-level change over time per se. We believe the trajectory-based approach (GBTM) is appropriate for this research question, as it captures heterogeneity in testing patterns and allows group-level comparisons using Cox regression, which directly addresses our primary aim.

That said, we acknowledge that we did not explicitly report within-person changes from baseline to end of follow-up. To address this concern without introducing additional statistical models that fall outside our pre-specified analysis plan, we have:

1.Added a limitation statement in the Limitation section acknowledging that we did not apply mixed-effects or GEE models to formally test within-person change, while noting that GBTM inherently captures individual-level longitudinal patterns by grouping participants who follow similar developmental trajectories.

2.Added descriptive comparisons of key outcomes between baseline and follow-up in the Discussion to provide readers with a general sense of observed changes.

(See Limitation in the revised manuscript, lines 27-35, page 10)

*Reviewer #1, Comment 2: *The manuscript reports recruitment between September 2021 and February 2022, with follow-up completed by February 2023, but elsewhere states that follow-up lasted “until March 2022.” These statements are inconsistent.*

Response: We apologize for this inconsistency. We have clarified the study timeline as follows:

Baseline recruitment: September 1, 2021 to February 28, 2022

Follow-up schedule: 3, 6, 9, and 12 months after baseline

Final follow-up completion: February 28, 2023

Maximum follow-up duration: 12 months for all participants

The inconsistent statement has been corrected.

(See Methods in the revised manuscript, lines 10-14, page 4)

*Reviewer #1, Comment 3: *The study period overlaps with COVID-19 control measures in China, which may have affected access to HIV testing and PrEP services.*

Response: We agree with the reviewer. We have added a discussion of this limitation in the Limitation section, noting that while study sites did not experience strict lockdowns during recruitment, local mobility restrictions and temporary clinic closures may have affected access to services. We have explicitly stated that readers should interpret the findings with consideration of this unique pandemic context.

(See Limitations in the revised manuscript, lines 8-14, page 10)

*Reviewer #1, Comment 4: The definitions of “low,” “middle,” and “high” HIV testing frequency are inconsistent between the main text and figure legend. The rationale for the chosen cut-points should be justified.

Response: We thank the reviewer for catching this inconsistency. We have:

1.Harmonized the definitions across the manuscript based on recommendations from multiple international guidelines: the WHO (2019) recommends annual testing for sexually active MSM and 3–6 monthly testing for those at increased risk; the US CDC (2025) recommends annual screening for MSM, with more frequent testing (every 3–6 months) for high-risk individuals; and the IAS-USA (2024) advises 6-monthly HIV testing for PrEP users and 3-monthly testing for frequently exposed individuals.

2.Final definitions: Low (<1 test/3 months), Medium (1–2 tests/3 months), and High (≥3 tests/3 months).

3.Added justification in the Methods section and updated the Figure 1 legend accordingly.

(See Methods in the revised manuscript, lines 3-10, page 5, and Fig 1 legend)

*Reviewer #1, Comment 5: The sample is relatively young and includes a high proportion of students, which may not fully represent the broader MSM population in China.

Response: We agree with the reviewer. We have:

1.Added comparisons with other Chinese MSM cohorts in the Limitations section: a cohort study in Zhuhai reported that MSM aged 18–24 accounted for 23.18%, with students comprising only 6.62%; a 28-province study (N= 2,618 ) reported 26.90% aged <25 years and 18.80% students; and a Northwest China study reported 16.3% aged 18–25 years with <8.1% students.

2.Explicitly stated that these comparisons suggest our sample is significantly younger and more student-dominated than many other Chinese MSM cohorts, limiting generalizability to older MSM and non-student populations.

3.Called for future studies with more diverse age and occupational groups to validate our findings.

(See Limitation in the revised manuscript, lines 15-24, page 10)

*Reviewer #1, Comment 6: The terms “pretreatment” and “post-treatment” visits are unclear in the context of this cohort analysis and may imply an intervention effect.

Response: These terms have been replaced with "baseline" and "follow-up" throughout the manuscript.

(See Methods in the revised manuscript, lines 24-25, page 4)

*Reviewer #1, Comment 7: *The authors may consider discussing digital tools in improving HIV testing, such as doi:10.1001/jamanetworkopen.2025.44821*

Response: We thank the reviewer for this suggestion. We have added a brief discussion on digital tools in improving HIV testing among MSM in the Discussion section, citing the recommended reference (doi:10.1001/jamanetworkopen.2025.44821). We note that integrating such digital approaches into HIV prevention programs in southern China may complement facility-based testing and help address barriers such as stigma and privacy concerns.

(See Discussion in the revised manuscript, lines 1-6, page 9, and Reference 36)

*Reviewer #1, Comment 8: *Table 1 appears to contain potential internal inconsistencies (e.g., reporting zero sexual partners while also reporting a high proportion of HIV-positive partners).*

We thank the reviewer for carefully identifying this issue. We sincerely apologize for this error. Upon re-examination, we found that the inconsistencies in Table 1 were caused by data transcription errors during the preparation of the manuscript. Specifically:

The variable "condomless anal sex with men (past 6 months)" was incorrectly placed: the correct values should be Yes: 182 (55.66%), No: 145 (44.34%). These data were previously assigned to the wrong row.

The variable "positive sex partner in the past 6 months" was also misclassified. The correct values are Yes: 43 (13.15%), No: 230 (70.34%), Uncertain: 54 (16.51%).

We have now carefully verified all data entries in Table 1 against the original dataset and corrected the erroneous row placements. The revised Table 1 has been updated accordingly, and all corrections have been highlighted in the revised manuscript.

To prevent such errors in the future, we have implemented a double-check procedure: all tables will now be independently verified by a second author against the original data before submission.

We sincerely apologize for these oversights and appreciate the reviewer's diligence in catching these issues.

(See Table 1)

Responses to Reviewer #2

*Reviewer #2 (General Comment): Very nicely laid out and written manuscript. Would revise to focus on 'Use of PrEP' among trajectories as primary objective and STI as secondary. The only technical concern is that some of the subgroups trajectories fall below 5% of the study population which may indicate that alternative grouping strategies need to be considered. Conclusion should also reconcile differences between PrEP and PEP uptake.

Response: Thank you for the positive feedback. We have:

1.Refocused the manuscript to present PrEP/PEP use as the primary outcome and STI as secondary, with adjustments made to the Abstract, Introduction, Methods, and Results sections.

2.Addressed the <5% subgroup issue by adding an explanation that although the 4.8% and 4.91% proportions are slightly below the pre-specified 5% threshold, sensitivity analyses with alternative model specifications (2-group and 4-group solutions) confirmed the presence of these distinct trajectory groups. The original 3-group solution was retained because it provided the best fit to the data (lowest BIC) and clinical interpretability.

3.Revised the Conclusion to separately discuss PrEP and PEP: PrEP use was more strongly associated with HIV self-testing, while PEP use was more closely linked to facility-based testing.

(See Introduction in the revised manuscript lines 42-43, page 3, Results lines 3-13, page 8, and Conclusion lines 39-43, page 10)

Reviewer #2 (Abstract Line #17): "men" not "man"

Response: Corrected. "man who have sex with man" has been changed to "men who have sex with men (MSM)" throughout the manuscript.

Reviewer #2 (Introduction): "INTRODUCTION" not "INTRUDITION"

Response: Corrected.

*Reviewer #2 (Introduction Lines 18-19): I believe that authors wanted to state that "retest" rates were less than 20%*

Response: We have clarified this statement: "An Australian study indicated that HIV retest rates among MSM were below 40% after one year and below 20% after six months."

Reviewer #2 (Introduction Line 42 & Methods Line 26): Aim in examining STI incidence is not consistent with abstract.

Response: We have revised these sentences to clarify that PrEP/PEP use is the primary objective and STI incidence is secondary.

(See Introduction in the revised manuscript lines 42-43, page 3, Results lines 3-13, page 8, and Conclusion lines 39-43, page 10)

Reviewer #2 (Statistics Line 8): Please include actual and minimum allowable posterior assignment probability.

Response: We have added: "the minimum acceptable posterior assignment probability for trajectory group membership was set at ≥0.8."

(See Methods in the revised manuscript, lines 17-18, page 5)

Reviewer #2 (Results Line 4 and 11): Please explain why high use of 4.8% and 4.91% were allowed when 5% was minimum.

Response: We have added an explanation in the Statistics section: "Although our pre-specified minimum group size was 5%, the 4.8% and 4.91% proportions represent slight deviations due to model optimization based on BIC. We conducted sensitivity analyses with alternative model specifications (2-group and 4-group solutions), which confirmed the presence of distinct trajectory groups. The original 3-group solution was retained because it provided the best fit to the data (lowest BIC) and clinical interpretability."

(See Methods in the revised manuscript, lines 18-23, page 5)

Reviewer #2: Would reorder Incidence of STI and Use of HIV Exposure Prophylaxis actions.

Response: The Results section has been reordered as suggested: "Use of HIV exposure prophylaxis (PrEP/PEP)" now appears before "Incidence and risk of STI."

(See Results in the revised manuscript, lines 7-13, page 8)

Reviewer #2 (Discussion and Conclusion): Statistics on condom use should be highlighted earlier; age and PEP association should be discussed.

Response: We have:

1.Added condom use descriptive statistics in the baseline characteristics section of the Results.

2.Added a discussion of potential barriers to linking self-testing to PEP for younger MSM: lack of awareness about PEP availability, perceived low risk, cost concerns, and fear of disclosure.

(See Results in the revised manuscript, lines 12-13, page 6, and Discussion lines 1-6, page 9)

Conclusion

We sincerely thank the editor and both reviewers for their time, expertise, and constructive feedback, which have been invaluable in strengthening our manuscript. We have made every effort to address all comments thoroughly and believe that the revised version is substantially improved.

We acknowledge that some of the methodological concerns raised—particularly regarding within-person change analyses—represent important avenues for future research. We are fully open to making additional revisions should the editor or reviewers feel that further improvements are needed. Please do not hesitate to let us know if any aspect of our response or revision requires additional clarification or adjustment; we would be happy to address it promptly.

We appreciate your continued consideration of our work and look forward to your further guidance.

Sincerely,

Ning Chuanyi, MD, PhD

Corresponding author

On behalf of all authors

Attachment Submitted filename: Response to Reviewers.docx

# Peer review: Decision Letter 1

**DOI:** 10.1371/journal.pone.0358578.r003

**Authors:** Long Nguyen

8 Jul 2026

Exploring the Relationship Between HIV Testing Frequency and Sexual Behavior, Prevention Prophylaxis Use Among MSM in Southern China

PLOS One

Dear Dr. Ning,

Please see the reviewers' comments and address them carefully.

==============================

Please submit your revised manuscript by Aug 22 2026 11:59PM. If you will need more time than this to complete your revisions, please reply to this message or contact the journal office at plosone@plos.org. When you're ready to submit your revision, log on to https://www.editorialmanager.com/pone/ and select the 'Submissions Needing Revision' folder to locate your manuscript file.

- A letter that responds to each point raised by the academic editor and reviewer(s). You should upload this letter as a separate file labeled 'Response to Reviewers'.
- A marked-up copy of your manuscript that highlights changes made to the original version. You should upload this as a separate file labeled 'Revised Manuscript with Track Changes'.
- An unmarked version of your revised paper without tracked changes. You should upload this as a separate file labeled 'Manuscript'.

If you would like to make changes to your financial disclosure, please include your updated statement in your cover letter. Guidelines for resubmitting your figure files are available below the reviewer comments at the end of this letter.

If applicable, we recommend that you deposit your laboratory protocols in protocols.io to enhance the reproducibility of your results. Protocols.io assigns your protocol its own identifier (DOI) so that it can be cited independently in the future. For instructions see: https://journals.plos.org/plosone/s/submission-guidelines#loc-laboratory-protocols. Additionally, PLOS ONE offers an option for publishing peer-reviewed Lab Protocol articles, which describe protocols hosted on protocols.io. Read more information on sharing protocols at https://plos.org/protocols?utm_medium=editorial-email&utm_source=authorletters&utm_campaign=protocols.

As the corresponding author, your ORCID iD is verified in the submission system and will appear in the published article. PLOS supports the use of ORCID, and we encourage all coauthors to register for an ORCID iD and use it as well. Please encourage your coauthors to verify their ORCID iD within the submission system before final acceptance, as unverified ORCID iDs will not appear in the published article. *Only*  the individual author can complete the verification step; PLOS staff *cannot*  verify ORCID iDs on behalf of authors.

We look forward to receiving your revised manuscript.

Kind regards,

Long Hoang Nguyen

Academic Editor

PLOS One

Journal Requirements:

If the reviewer comments include a recommendation to cite specific previously published works, please review and evaluate these publications to determine whether they are relevant and should be cited. There is no requirement to cite these works unless the editor has indicated otherwise.

[Note: HTML markup is below. Please do not edit.]

Reviewers' comments:

Reviewer's Responses to Questions

**Comments to the Author**

Reviewer #1: All comments have been addressed

Reviewer #3: (No Response)

**********

2. Is the manuscript technically sound, and do the data support the conclusions?

Reviewer #1: Yes

Reviewer #3: Yes

**********

3. Has the statistical analysis been performed appropriately and rigorously?

Reviewer #1: Yes

Reviewer #3: Yes

**********

4. Have the authors made all data underlying the findings in their manuscript fully available?

The PLOS Data policy

Reviewer #1: Yes

Reviewer #3: No

**********

5. Is the manuscript presented in an intelligible fashion and written in standard English?

Reviewer #1: Yes

Reviewer #3: Yes

**********

Reviewer #1: Thank you for inviting me to re-review the revised manuscript. All the comments have been addressed.

Reviewer #3: The authors have addressed most of the concerns raised in the previous review, and the manuscript has improved substantially in clarity, organization, and interpretation. The additional discussion on COVID-19, generalizability, digital HIV testing tools, and the rationale for the trajectory groups strengthens the manuscript. The revised analyses and responses are generally satisfactory.

However, several issues remain that should be corrected before the manuscript is accepted. Most are issues of consistency, completeness, and presentation rather than fundamental methodological concerns.

Comments to the Authors

Thank you for carefully revising the manuscript and providing detailed responses to the reviewers. I appreciate the considerable effort made to address the previous comments. I have only a few remaining suggestions.

1. The definitions of low-, middle-, and high-frequency HIV testing remain inconsistent between the Methods section and Figure 1. In the Methods, the categories are defined as low (<1 test/3 months), middle (1–2 tests/3 months), and high (≥3 tests/3 months). However, the Figure 1 legend still defines the middle group as "1 time every 3 months" and the high group as "≥2 times every 3 months." These definitions should be fully harmonized throughout the manuscript.

2. In the response letter, the authors state that descriptive comparisons between baseline and follow-up outcomes were added. However, I could not clearly identify these comparisons in the revised manuscript. Either include the promised descriptive comparison or revise the response letter to accurately reflect the changes that were made.

3. The response letter indicates that discussion was added regarding barriers to PEP uptake among younger MSM (e.g., awareness, perceived risk, cost, disclosure concerns). I was unable to locate this discussion in the revised manuscript. Please either incorporate the promised discussion or modify the response accordingly.

4. The manuscript states that sensitivity analyses using alternative trajectory models (2-group and 4-group solutions) confirmed the selected model and refers readers to Supplementary Table S1. Please ensure that this supplementary table is included in the submission and contains the reported model fit statistics.

Minor inconsistencies and typographical errors

Several editorial issues remain and should be corrected during revision, including:

Figure 1 legend incorrectly describes HIVST as "HIV facility-based testing."

The heading "Use of HIV exposure prophylaxis prophylaxis" contains a duplicated word.

Multiple duplicated punctuation marks (e.g., "..").

Minor spacing and formatting inconsistencies.

The sentence beginning "WHO has HIVST serves as..." is grammatically incorrect and should be rewritten.

The statement "Sequence data have been deposited..." is inappropriate for this study and appears inconsistent with the revised Data Availability Statement. Please replace it with language consistent with the actual data-sharing policy.

Consistency between the response letter and manuscript

A few statements in the response letter describe revisions that are not fully reflected in the manuscript. Before resubmission, I recommend carefully cross-checking the response letter against the revised manuscript to ensure that every promised revision has actually been implemented.

**********

what does this mean?). If published, this will include your full peer review and any attached files.

If you choose “no”, your identity will remain anonymous but your review may still be made public.

**Do you want your identity to be public for this peer review?**  For information about this choice, including consent withdrawal, please see our Privacy Policy

Reviewer #1: No

Reviewer #3: No

**********

[NOTE: If reviewer comments were submitted as an attachment file, they will be attached to this email and accessible via the submission site. Please log into your account, locate the manuscript record, and check for the action link "View Attachments". If this link does not appear, there are no attachment files.]

To ensure your figures meet our technical requirements, please review our figure guidelines: https://journals.plos.org/plosone/s/figures

You may also use PLOS’s free figure tool, NAAS, to help you prepare publication quality figures: https://journals.plos.org/plosone/s/figures#loc-tools-for-figure-preparation.

NAAS will assess whether your figures meet our technical requirements by comparing each figure against our figure specifications.

# Author comment: Author response to Decision Letter 2

**DOI:** 10.1371/journal.pone.0358578.r004

11 Jul 2026

Manuscript ID: PONE-D-25-58406R1

Title: Exploring the Relationship Between HIV Testing Frequency and Sexual Behavior, Prevention Prophylaxis Use Among MSM in Southern China

Journal: PLOS ONE

Dear Dr. Nguyen and Reviewers,

Thank you very much for giving us the opportunity to revise and resubmit our manuscript to PLOS ONE. We are sincerely grateful to the reviewers for taking the time to carefully evaluate our work. The constructive comments have been invaluable in strengthening our manuscript. We have addressed all concerns point-by-point as detailed below. All changes in the manuscript have been highlighted using track changes.

Responses to Reviewer #3

*Reviewer #3, Comment 1: The definitions of low-, middle-, and high-frequency HIV testing remain inconsistent between the Methods section and Figure 1. In the Methods, the categories are defined as low (<1 test/3 months), middle (1–2 tests/3 months), and high (≥3 tests/3 months). However, the Figure 1 legend still defines the middle group as “1 time every 3 months” and the high group as “≥2 times every 3 months.”

Response: We thank the reviewer for this careful observation. We have now thoroughly harmonized the definitions across the entire manuscript. The final definitions are: Low frequency (<1 test per 3 months), Medium frequency (1–2 tests per 3 months), and High frequency (≥3 tests per 3 months). These definitions have been consistently applied in the Methods section, Results section, Fig 1 (main text and legend). All corresponding text and figure legends have been revised and highlighted in the updated manuscript.

(See Results section: "Definition of Trajectory Groups"; Fig 1 legend and Fig 1)

*Reviewer #3, Comment 2: In the response letter, the authors state that descriptive comparisons between baseline and follow-up outcomes were added. However, I could not clearly identify these comparisons in the revised manuscript.

Response: We thank the reviewer for this comment. In the previous revision, we added descriptive comparisons between baseline and follow-up outcomes in the Discussion section, specifically within the paragraph on descriptive trends. To present these data more clearly, we have also provided the full descriptive comparison table as Supplementary Table S3. This table includes key indicators such as HIV testing frequency (overall, HIVST, and SBHT), PrEP/PEP use, unprotected sexual behavior, and STI cases from baseline to each follow-up time point.

We have noted in both the table and the Discussion that baseline recall periods differ from follow-up: HIV testing reflects the past 12 months at baseline versus the past 3 months at follow-up; unprotected sexual behavior reflects the past 6 months at baseline versus the past 3 months at follow-up; and PrEP/PEP use reflects lifetime use at baseline versus past-3-month use at follow-up. These descriptive data are intended to provide readers with an overview of raw trends and no formal statistical testing was performed.

(See Discussion section, lines 24-34, page 8; Supplementary sTable3)

*Reviewer #3, Comment 3: The response letter indicates that discussion was added regarding barriers to PEP uptake among younger MSM. I was unable to locate this discussion in the revised manuscript.

Response: We thank the reviewer for pointing this out. We apologize for this omission. We have now added a discussion of barriers to PEP uptake among younger MSM in the Discussion section. We note that there is a gap between frequent HIV self-testing among younger MSM and low PEP uptake. Potential barriers include limited awareness of PEP access, perceived low risk despite frequent testing, out-of-pocket cost concerns, and fear of stigma when seeking PEP at clinics. These findings suggest that integrating PEP education into self-testing platforms and establishing rapid referral pathways may help bridge this gap, particularly for younger MSM.

(See Discussion section, lines 11-23, page 10)

*Reviewer #3, Comment 4: The manuscript states that sensitivity analyses using alternative trajectory models confirmed the selected model and refers readers to Supplementary Table S1. Please ensure that this supplementary table is included in the submission.

Response: We confirm that Supplementary Table S1 has been included in the revised submission. This table contains the model fit assessment indicators (BIC, AIC, and posterior assignment probabilities) for all trajectory models. The table is referenced in the Methods section (Statistical Analysis paragraph) and has been uploaded as part of the Supporting Information files.

(See Supplementary sTable1)

*Reviewer #3 (Minor inconsistencies and typographical errors):

Response: We have carefully corrected all editorial issues identified by the reviewer:

Fig 1 legend: Corrected the erroneous description from "(C) HIVST: HIV facility-based testing" to "(C) HIVST: HIV self-testing."

Duplicate words: Corrected "Use of HIV exposure prophylaxis prophylaxis" to "Use of HIV exposure prophylaxis" in the Results section heading.

Duplicate punctuation: Removed all instances of double periods ("..") throughout the manuscript.

Spacing and formatting: Corrected minor spacing and formatting inconsistencies throughout the manuscript.

Grammatical error: Rewrote "The World Health Organization (WHO) has HIVST serves as a strategy..." to "The World Health Organization (WHO) recommends HIVST as a strategy to enhance the accessibility of HT for individuals at elevated risk."

Data Availability Statement: Replaced the inappropriate statement with the correct Data Availability Statement.

*Reviewer #3 (Consistency between the response letter and manuscript):

Response: We sincerely thank the reviewer for this important reminder. We have carefully cross-checked every statement in the response letter against the actual revised manuscript. All promised revisions have been implemented and highlighted in the revised manuscript with track changes.

Journal Requirements

Response to Data Availability Statement: We have updated the Data Availability Statement to comply with PLOS ONE's policy. The revised statement reads:

"The raw data supporting the findings of this study are not publicly available due to the sensitive nature of the information collected (including sexual behavior and HIV status), which could compromise participant privacy. Data requests may be directed to the Institutional Review Board at Guangxi Medical University (gxmuyfyll@163.com), which will review requests for data access in accordance with the ethical approval granted for this study."

(See Methods section, lines 39-43, page 5)

Conclusion

We sincerely thank the editor and reviewers for their valuable time and expert guidance in improving our manuscript. Each comment has been deeply insightful and has contributed significantly to strengthening our work. We have made every effort to address all comments thoroughly and believe that the revised version is substantially improved in both academic rigor and completeness of presentation.

We fully recognize that scholarly writing is an ongoing process. Should the editor or reviewers feel that further improvements are needed, we remain entirely open to making additional revisions. Please do not hesitate to let us know if any aspect of our response or revision requires further clarification or adjustment; we would be happy to address it promptly.

We sincerely appreciate your continued consideration of our work and look forward to your final decision.

Sincerely,

Chuanyi Ning, MD, PhD

Corresponding author

On behalf of all authors

Attachment Submitted filename: Response to Reviewers 2.docx

# Peer review: Decision Letter 2

**DOI:** 10.1371/journal.pone.0358578.r005

**Authors:** Yury Khudyakov

2 Sep 2026

Exploring the Relationship Between HIV Testing Frequency and Sexual Behavior, Prevention Prophylaxis Use Among MSM in Southern China

PONE-D-25-58406R2

Dear Dr. Ning,

We’re pleased to inform you that your manuscript has been judged scientifically suitable for publication and will be formally accepted for publication once it meets all outstanding technical requirements.

Within one week, you’ll receive an e-mail detailing the required amendments. When these have been addressed, you’ll receive a formal acceptance letter and your manuscript will be scheduled for publication.

An invoice will be generated when your article is formally accepted. Please note, if your institution has a publishing partnership with PLOS and your article meets the relevant criteria, all or part of your publication costs will be covered. Please make sure your user information is up-to-date by logging into Editorial Manager at Editorial Manager® and clicking the ‘Update My Information' link at the top of the page. For questions related to billing, please contact billing support.

If your institution or institutions have a press office, please notify them about your upcoming paper to help maximize its impact. If they’ll be preparing press materials, please inform our press team as soon as possible -- no later than 48 hours after receiving the formal acceptance. Your manuscript will remain under strict press embargo until 2 pm Eastern Time on the date of publication. For more information, please contact onepress@plos.org.

Kind regards,

Yury E Khudyakov, PhD

Academic Editor

PLOS One

Additional Editor Comments (optional):

Reviewers' comments:

Reviewer's Responses to Questions

**Comments to the Author**

Reviewer #1: All comments have been addressed

**********

2. Is the manuscript technically sound, and do the data support the conclusions?

Reviewer #1: Yes

**********

3. Has the statistical analysis been performed appropriately and rigorously?

Reviewer #1: Yes

**********

4. Have the authors made all data underlying the findings in their manuscript fully available?

The PLOS Data policy

Reviewer #1: Yes

**********

5. Is the manuscript presented in an intelligible fashion and written in standard English?

Reviewer #1: Yes

**********

Reviewer #1: All the comments have been addressed. Thank you for inviting me to have a review of the revised mansucript.

**********

what does this mean?). If published, this will include your full peer review and any attached files.

If you choose “no”, your identity will remain anonymous but your review may still be made public.

**Do you want your identity to be public for this peer review?**  For information about this choice, including consent withdrawal, please see our Privacy Policy

Reviewer #1: No

**********

# Editor report: Acceptance letter

**DOI:** 10.1371/journal.pone.0358578.r006

**Authors:** Yury Khudyakov

PONE-D-25-58406R2

PLOS One

Dear Dr. Ning,

I'm pleased to inform you that your manuscript has been deemed suitable for publication in PLOS One. Congratulations! Your manuscript is now being handed over to our production team.

At this stage, our production department will prepare your paper for publication. This includes ensuring the following:

* All references, tables, and figures are properly cited

* All relevant supporting information is included in the manuscript submission,

* There are no issues that prevent the paper from being properly typeset

You will receive further instructions from the production team, including instructions on how to review your proof when it is ready. Please keep in mind that we are working through a large volume of accepted articles, so please give us a few days to review your paper and let you know the next and final steps.

Lastly, if your institution or institutions have a press office, please let them know about your upcoming paper now to help maximize its impact. If they'll be preparing press materials, please inform our press team within the next 48 hours. Your manuscript will remain under strict press embargo until 2 pm Eastern Time on the date of publication. For more information, please contact onepress@plos.org.

You will receive an invoice from PLOS for your publication fee after your manuscript has reached the completed accept phase. If you receive an email requesting payment before acceptance or for any other service, this may be a phishing scheme. Learn how to identify phishing emails and protect your accounts at https://explore.plos.org/phishing.

If we can help with anything else, please email us at customercare@plos.org.

Thank you for submitting your work to PLOS One and supporting open access.

Kind regards,

PLOS One Editorial Office Staff

on behalf of

Dr. Yury E Khudyakov

Academic Editor

PLOS One
